# Organoids, organ-on-a-chip, and microtumors: Biomimetic 3D tumor models advancing drug development and precision medicine

**DOI:** 10.1016/j.apsb.2026.03.013

**Published:** 2026-03-12

**Authors:** Mengjiao Xia, Guohua Wu, Di Wu, Wenqi Hu, Hongbin Deng, Shuqi Wang

**Affiliations:** a Clinical Research Center for Respiratory Disease, West China Hospital, Sichuan University, Chengdu 610041, China; b College of Biomedical Engineering, Sichuan University, Chengdu 610065, China; c National Engineering Research Center for Biomaterials, Sichuan University, Chengdu 610065, China; d Tianfu Jincheng Laboratory, City of Future Medicine, Chengdu 641400, China; e Luoyang Key Laboratory of Clinical Multiomics and Translational Medicine, Henan Key Laboratory of Rare Diseases, Endocrinology and Metabolism Center, The First Affiliated Hospital, and College of Clinical Medicine of Henan University of Science and Technology, Luoyang 471003, China; f Department of Respiratory and Critical Care Medicine, West China Hospital, Sichuan University, Chengdu 610041, China; g State Key Laboratory of Respiratory Health and Multimorbidity, West China Hospital, Sichuan University, Chengdu 610041, China; h Institute of Medicinal Biotechnology, Chinese Academy of Medical Sciences and Peking Union Medical College, Beijing 100050, China

**Keywords:** Organoids, Organ-on-a-chip, Microtumors, Drug screening, Precision oncology, Personalized therapy, Tumor microenvironment, Artificial intelligence

## Abstract

The rising global cancer burden underscores the urgent need for more effective drug development and personalized therapies. Conventional screening models, such as 2D cell lines and patient-derived xenografts, fail to adequately recapitulate the architecture, heterogeneity, and microenvironment of human tumors, limiting their clinical translatability. In response, human-derived biomimetic platforms have emerged. Organoids and organ-on-a-chip preserve key tumor genetics and stimulate dynamic, physiologically relevant microenvironments, whereas microtumors are distinguished by high biological fidelity. Microtumors uniquely retain the native tumor ecosystem, capture a broader spectrum of intratumoral heterogeneity, and, critically, maintain a functional immune microenvironment. Together, these systems enable drug screening that more faithfully reflects the clinical context, with strong potential to raise drug development success rates and support individualized therapy. This review consolidates the cutting-edge advancements and critical challenges associated with these models in drug development, precision medicine, and clinical translation. Furthermore, it envisions how Artificial Intelligence (AI) can drive its intelligent evolution, aiming to provide a robust evidentiary basis and practical reference for research and clinical practice, thereby propelling the field of precision oncology into a new era.

## Graphical abstract

Biomimetic 3D models, including organoids, organ-on-a-chip, and microtumors, recapitulate the patient-specific tumor microenvironment to advance drug development and precision medicine.

**Figure:**
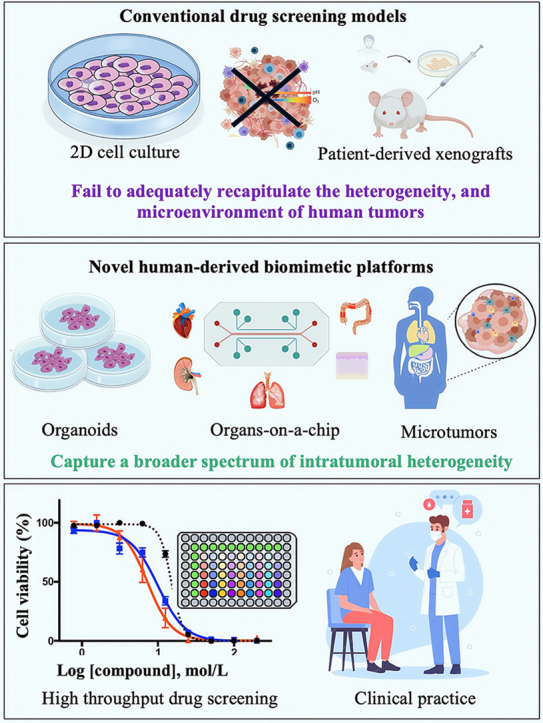

## 1 Introduction

The International Agency for Research on Cancer reports that in 2022, there were approximately 20 million new cancer cases and 9.7 million deaths, and that new cases will rise to 35 million by 2050, posing a substantial challenge to global public health systems^1^^,^^2^. In China, data from the National Cancer Center reveal a comparable trend, with approximately 4.82 million new cancer cases (22.7% of the global total) and 2.57 million deaths (26.7% of the global total) in 2022, owing to factors such as population aging, lifestyle changes, and environmental exposures^3^. These statistics underscore that cancer is placing a substantial strain on healthcare resources, socioeconomic development, and patient well-being around the world. A pivotal issue regarding cancer diagnosis and treatment is tumor heterogeneity, a phenomenon characterized by the profound variations in the genomic, epigenetic, and microenvironmental landscapes that exist both within and between tumors^4^^,^^5^. Particularly, tumor heterogeneity leads to a wide spectrum of therapeutic responses to a certain cancer. For example, in advanced non-small cell lung cancer, the response rate to targeted therapy exceeds 70% for patients with epidermal growth factor receptor (*EGFR*) mutations but is less than 10% for wild-type patients^6^. Consequently, developing personalized treatment strategies is a clinical imperative for improving survival rates^4^^,^^5^^,^^7^. However, tumor heterogeneity presents several clinical challenges: (1) Treatment: Standardized, one-size-fits-all protocols based on population-level data often fail to align with the specific biological characteristics of an individual's tumor. For instance, while The Cancer Genome Atlas data identifies at least 10 molecular subtypes of breast cancer, standard clinical protocols address only four^6^^,^^8^. (2) Drug resistance and recurrence: Heterogeneity drives clonal evolution, leading to the coexistence of drug-sensitive and drug-resistant subclones. The detection of a *KRAS*-mutated subclone in post-surgical residual disease of a colorectal cancer (CRC) patient, for example, was associated with relapse within six months^4^^,^^5^. (3) Efficacy prediction: The predictive power of traditional drug screening models, such as cell lines and animal models, is limited by their inability to fully recapitulate the dynamic interactions of the human tumor microenvironment (TME)^4^, thereby impeding therapeutic advancement.

Conventional drug screening platforms predominantly consist of immortalized cell lines, traditional animal models, and patient-derived xenograft (PDX) models, each of which exhibits significant limitations^9^, ^10^, ^11^. (1) Immortalized cell lines: Prolonged *in vitro* cultivation leads to genotypic and phenotypic drift. For instance, glioblastoma cell lines progressively lose their differentiation capacity when cultured *in vitro*, failing to recapitulate the intrinsic heterogeneity of tumors^12^. Moreover, the 2D culture format cannot adequately replicate the dynamic interactions within the TME among cells, ECM, immune cells, and cytokines^9^, ^10^, ^11^, thereby diminishing its predictive clinical value^13^. (2) Conventional animal models: Despite their widespread historical use, partly driven by regulatory policies from agencies such as the U.S. Food and Drug Administration (FDA), their utility is compromised by interspecies differences that can lead to misleading conclusions^14^^,^^15^. Research indicates that over 90% of drugs deemed effective in animal studies ultimately fail in human clinical trials, contributing to an average loss of $64 million and 5–10 years in development time per compound^14^, ^15^, ^16^. This profound translational chasm has spurred regulatory shifts, exemplified by the FDA's recent initiatives to phase out mandatory animal testing for certain pharmaceuticals, such as monoclonal antibodies^17^. (3) PDX models: Generated by engrafting patient tumor fragments into immunodeficient hosts, typically preserve 85% genomic concordance with their donor tumors. Across cohorts of advanced solid cancers, overall initial engraftment rates around 49% have been reported, with stable line establishment somewhat lower and dependent on tumor type, stage, host strain, and sample handling. However, their establishment is a protracted process, requiring 3–6 months^11^^,^^18^^,^^19^. For patients with advanced cancer, who often have a median survival of less than 12 months, this extended timeline directly precludes 20%–40% of them from receiving timely, model-informed therapeutic guidance (Table 1)^11^^,^^13^, ^14^, ^15^, ^16^^,^^19^, ^20^, ^21^, ^22^, ^23^.

**Table 1:** A comparative analysis of core limitations in traditional drug screening models.

Model type	Deficiencies in precision	Timeliness deficiencies	Clinical decision suitability
Immortalized cell lines	Genotypic and phenotypic drift^19^^,^^20^; Loss of microenvironmental interactions in 2D culture^20^^,^^21^.	Rapid turnaround, but low predictive clinical value^13^^,^^20^.	Applicable primarily for preliminary screening.
Conventional animal models	High rate of false positives (>90%) due to interspecies differences^14^, ^15^, ^16^; Distortion of signaling pathways across species^21^	Moderate timeframe, but poor translation to clinical efficacy^11^	Limited clinical translatability due to interspecies differences^19^.
PDX models	Absence of a functional immune microenvironment^11^^,^^22^.	Protracted establishment period (3–6 months)^15^; Up to 40% of patients miss the therapeutic window^11^^,^^23^.	High genomic fidelity is offset by a critically slow timeline.

Conventional drug screening models are fundamentally limited by their lack of precision and timeliness, failing to meet the stringent requirements for rapid and accurate drug discovery in the era of personalized medicine. To bridge this gap, innovative platforms such as organoids, organs-on-chips, and microtumors leverage 3D biomimetic engineering and microfluidic control to circumvent these drawbacks. By dynamically recapitulating complex physiological functions and patient-specific pathologies, these emerging technologies provide novel platforms for tumor modeling, clinical drug screening, precision drug development, and personalized therapy^13^^,^^22^, ^23^, ^24^, ^25^, ^26^, ^27^.

**Figure 1:**
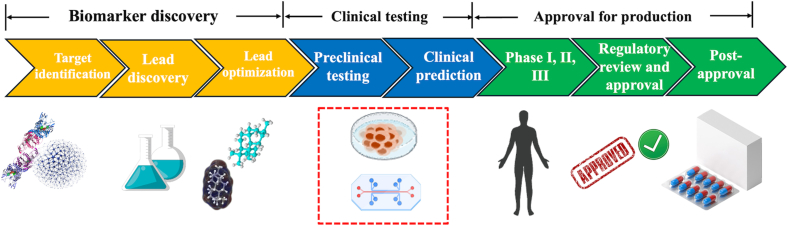
Positioning of biomimetic 3D tumor models on the timeline of drug development and clinical translation. Drug development and clinical translation are typically divided into three core components: (1) the discovery phase (yellow), covering target identification, lead compound discovery, and optimization, primarily driven by large-scale omics and computational methods; (2) the preclinical and clinical development phase (blue), conducting rigorous efficacy and safety testing of drug candidates; (3) the regulatory approval and post-marketing surveillance phase (green). The transition from preclinical testing to human clinical trials is a recognized translational bottleneck. Due to insufficient simulation of human physiological and pathological states by traditional models, such as animal models and two-dimensional (2D) cell lines, their predictive capability is limited, resulting in a large number of promising drug candidates failing at this critical stage due to unexpected toxicity or poor efficacy. The core argument of this review is that advanced biomimetic 3D tumor models, such as organ-on-a-chip, PDOs, and microtumors, are employed to address this translational bottleneck (Adapted from Ref. 33. Copyright © 2022 MDPI).

While this review provides a comprehensive overview of organoid, organ-on-a-chip, and microtumor technologies, its unique analytical perspective lies in the precise positioning of these models within the drug development and clinical translation timeline (Fig. 1). We emphasize that the primary utility of these advanced systems is not in the initial, de novo phases of target or biomarker discovery, which remain dominated by large-scale omics approaches^28^, ^29^, ^30^. Instead, their core value is realized downstream, in the critical phases of preclinical testing and clinical response prediction. This constitutes a well-documented translational bottleneck, where an estimated 90% of drug candidates entering clinical trials ultimately fail due to a lack of efficacy or unforeseen toxicity not predicted by traditional preclinical models^31^^,^^32^.

Accordingly, this review systematically elucidates how these platforms bridge this critical gap. Organ-on-a-chip systems are increasingly used to generate human-relevant pharmacokinetic and toxicity data, offering a powerful alternative to animal testing^34^. Patient-derived organoids (PDOs) serve as high-fidelity “patient avatars” for predicting therapeutic response^34^. While microtumors particularly preserve the native TME architecture, including patient-specific immune cells and stromal components, thereby enabling an authentic *ex vivo* assessment of TME-mediated response and resistance mechanisms to immunotherapies^35^. However, the successful translation of these models into routine clinical practice is not guaranteed. A central component of our review is therefore a critical assessment of the primary obstacles impeding their widespread adoption.

We analyze persistent challenges such as the lack of standardized protocols for culture and assaying, the substantial cost and labor intensity that limit throughput and scalability, and the evolving but yet-to-be-defined regulatory pathways for their use as decision-making tools^2^^,^^36^. This review posits that future breakthroughs will necessitate interdisciplinary fusion, such as AI-powered automation and scalable microfluidics. Based on this analysis, we outline a technology–clinical framework aimed at realizing the predictive potential of these models, ultimately providing a theoretical cornerstone for translational research that is both scientifically rigorous and clinically prescient.

## 2 Organoids, organ-on-a-chip, and microtumors

### 2.1 Organoids

#### 2.1.1 An overview of organoids

Organoids are microphysiological systems that arise within a three-dimensional (3D) culture environment from the self-organization of pluripotent stem cells (iPSCs), progenitor cells, or differentiated cells, facilitated by dynamic interactions with ECM^37^^,^^38^. They closely recapitulate the spatial architecture, cellular lineage differentiation, and functional activity of their organ of origin^39^, thereby offering an invaluable platform for developmental biology, disease modeling, and precision medicine^40^, ^41^, ^42^.

The field has evolved through three pivotal stages of advancement^22^^,^^42^. (1) Foundational discovery (2009–2013): In 2009, the group of Hans Clevers established the first self-organizing intestinal crypt-villus structures from *Lgr5+* stem cells. This seminal work overturned the long-held paradigm that tissue morphogenesis was inextricably dependent upon an *in vivo* developmental environment, laying the cornerstone for the entire field of organoid research^43^. This was followed in 2013 by the advent of cerebral organoids and functional liver bud organoids. The former successfully recapitulated synaptic transmission, providing a novel platform for interrogating the mechanisms of neurodevelopmental disorders^44^, while the latter, upon *in vivo* transplantation, achieved vascularization and sustained albumin secretion, confirming the successful reconstruction of metabolic functionality^45^. These milestones signified a significant advance in the ability to biomimetically model complex organs. (2) Expansion into precision oncology (2015–2018): Hans Clevers and his collaborators established comprehensive PDO biobanks, encompassing a spectrum of cancer types, including colorectal, breast, and gastric cancers^46^, ^47^, ^48^. These biobanks showcased the profound potential of organoids for predicting patient sensitivity to standard chemotherapeutic agents such as oxaliplatin and irinotecan, as well as for facilitating personalized therapeutic strategies and investigating tumor heterogeneity. (3) Standardization and clinical translation (2020–2025): an influential study introduced an organoid microsphere platform that demonstrated a remarkable 97% fidelity in preserving driver gene mutations and achieved a clinical prediction accuracy exceeding 80%^49^. Advancements in patterning techniques have enabled the fabrication of intestinal organoids with precisely controlled shapes, sizes, and cellular distributions, resulting in structures that more closely emulate native organ architecture. The high reproducibility of this approach has significantly propelled the field toward greater standardization^50^. Simultaneously, through the optimization of culture conditions, researchers have successfully engineered liver organoids that exhibit the metabolic functions of adult hepatocytes. Crucially, upon *in vivo* transplantation, these organoids have been shown to promote the regeneration of failing livers^51^, thereby presenting a viable therapeutic alternative for end-stage liver disease and accelerating the clinical translation of regenerative medicine^22^.

#### 2.1.2 A methodological workflow for organoid generation and validation

The generation of organoids is a multi-step process where the initial choice of biological starting material and its subsequent processing are critical determinants of the final model's fidelity and application scope. The workflow begins with a fundamental decision between two primary sources: patient-derived tissues, which yield adult stem cell (ASC)-driven organoids, and induced pluripotent stem cells (iPSCs)^23^. ASC-derived organoids, harvested directly from patient biopsies, are the preferred source for precision medicine. They inherently capture the patient-specific genomic, epigenetic, and mutational landscape of the primary tumor^47^. However, their establishment success rate is highly variable and tumor-type dependent, ranging from over 85% for CRC to as low as 42% for pancreatic cancer, often limited by the quality and quantity of the initial biopsy material^37^^,^^46^. Conversely, iPSCs offer experimental control and scalability. They can be generated from easily accessible somatic cells, such as fibroblasts or peripheral blood mononuclear cells, differentiated into various organoid types, and subjected to precise genome editing *via* CRISPR-Cas9 to investigate the function of specific mutations^52^. However, the reprogramming and differentiation processes may not fully recapitulate the epigenetic signatures or the aged cellular state of the original tumor, potentially affecting the model's predictive validity for age-related cancers^53^.

**Figure 2:**
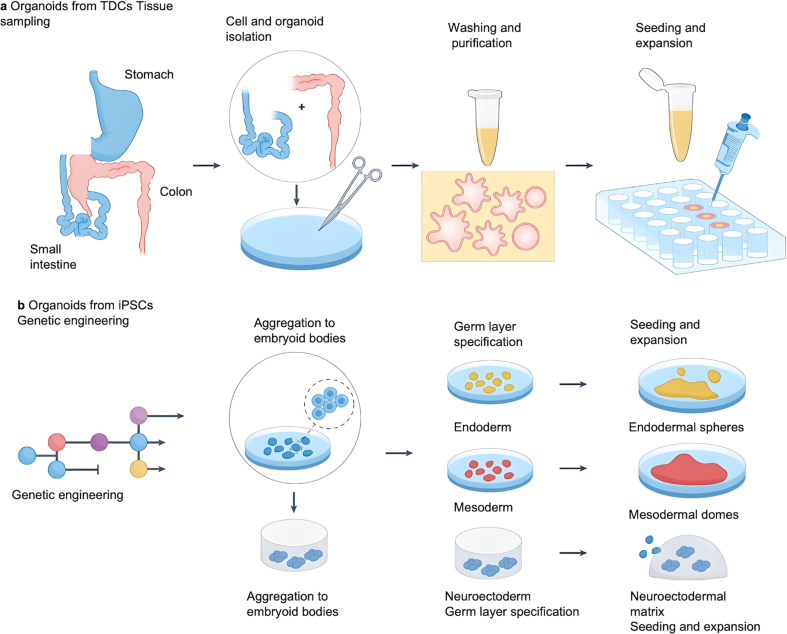
The generation of organoids *via* the expansion of tissue-derived stem cells and the directed differentiation of iPSCs^41^. (a) The process begins by mincing washed intestinal tissue samples into small fragments (2–4 mm). This increases the available surface area for subsequent enzymatic digestion or further mechanical dissociation, which facilitates the isolation of individual intestinal stem cells or intact crypts. Following several rounds of washing and purification, the harvested stem cells or crypts are seeded to establish and expand the organoid cultures. (b) For the directed generation of organoids from iPSCs, the initial phase of iPSC culture and expansion is of paramount importance. This process typically involves maintaining the iPSCs as undifferentiated colonies, either on a feeder-cell layer or on a substrate pre-coated with extracellular matrix (ECM) components, to facilitate their subsequent aggregation into embryoid bodies. Subsequently, the iPSCs are often harvested as cellular aggregates, a method that promotes robust cell-to-cell contact and yields a highly viable cell population. These aggregates are then subjected to directed differentiation protocols to specify germ layer identity, leading to the formation of structures such as endodermal spheroids, mesodermal domes, and neuroectodermal matrices for application in a diverse range of research studies (Adapted with permission from Ref. 41. Copyright © 2022 Springer Nature).

The standardized generation protocol comprises three crucial stages (Fig. 2). (1) Tissue dissociation: Patient-derived tissue, such as a tumor biopsy, is subjected to mechanical dissociation and subsequent enzymatic digestion with collagenase/trypsin to yield a suspension of single cells or micro-tissue fragments. This process is designed to comprehensively preserve the original genetic characteristics and pathological phenotype of the source tissue. (2) Matrix embedding: The resulting cells are embedded within an ECM analogue such as Matrigel, a basement membrane extract derived from Engelbreth-Holm-Swarm mouse sarcoma. It is rich in key ECM proteins like laminin and collagen IV, effectively supporting the growth of a wide variety of organoids^22^^,^^42^. (3) Directed induction: Cultured in a specialized medium, the cells undergo self-assembly to form organoids. This process relies on the precise recapitulation of the *in vivo* stem cell niche^54^. The medium is supplemented with key pathway modulators that maintain the cancer stem cell (CSC) population and drive hierarchical differentiation. For instance, the culture medium for CRC organoids is precisely formulated with several key components. Wnt agonists, such as *WNT3A* or *RSPO1*, are included to sustain the *LGR5+* CSC pool. *NOG* is used to inhibit BMP signaling, thereby preventing premature differentiation. *EGF* is added to promote proliferation^41^^,^^43^. The withdrawal or addition of specific factors can functionally probe tumor evolution. For example, a landmark study demonstrated that as CRC progresses, the derived organoids gradually lose their dependence on these niche factors, mirroring the acquisition of growth factor autonomy observed *in vivo*^55^^,^^56^. This highlights the model's capacity to not only maintain but also study the dynamics of cancer stemness^57^.

Organoids generated *via* this standardized protocol exhibit high fidelity, preserving key driver gene mutations of the primary tumor, such as *KRAS* and *TP53*, and maintaining genetic stability for over 20 passages^25^^,^^58^. Furthermore, they effectively recapitulate crucial aspects of the tumor, including cellular heterogeneity and stromal–epithelial interactions^39^. In the context of gastrointestinal cancer models, these organoids have demonstrated a drug sensitivity prediction accuracy of 88%. This performance provides a functional assessment of the resisting cell death hallmark, with one pivotal study reporting 100% sensitivity and 93% specificity in predicting patient clinical outcomes^59^. This accuracy significantly outperforms that of traditional cell lines, which is typically below 50%^25^. Coupled with a relatively short generation time (2–4 weeks) and their amenability to high-throughput screening of over 100 compounds simultaneously, these attributes underscore their profound value for clinical translation and application. In comparison to conventional animal models and 2D cell lines, PDOs have emerged as a transformative platform for oncological research and personalized medicine. PDOs are distinguished by their high fidelity in recapitulating the genetic and phenotypic characteristics of the primary tumor, including its mutational landscape (such as gene variations and copy number alterations), tissue architecture, cellular heterogeneity, and pharmacological responses^24^.

**Figure 3:**
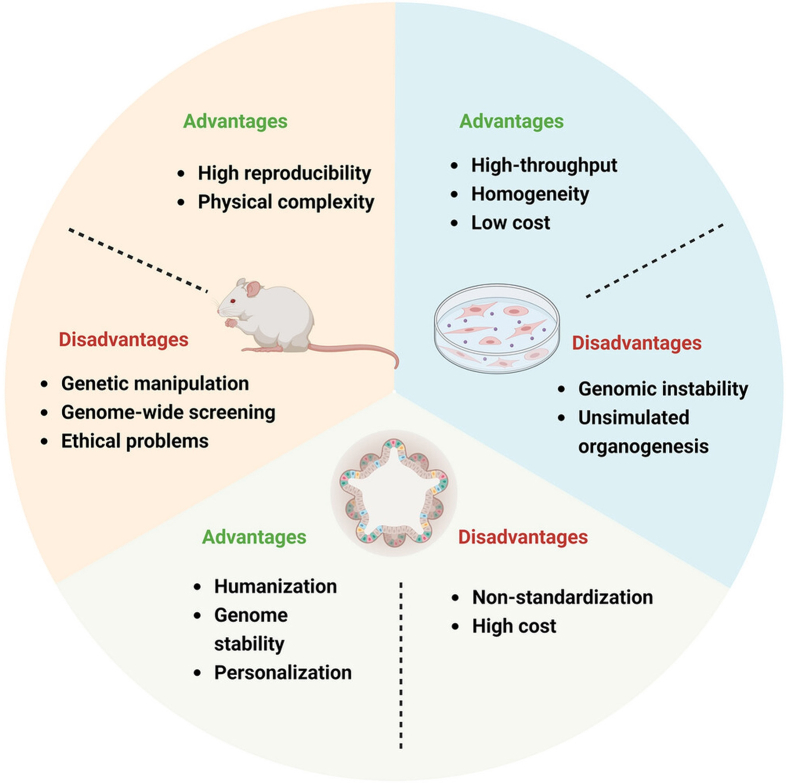
A comparative analysis of oncological research models^60^. Cell lines offer the distinct advantages of high-throughput capacity, sample homogeneity, and economic feasibility, yet their utility is curtailed by genomic instability and an inability to recapitulate the complex process of organogenesis. Animal models are distinguished by their exceptional reproducibility; however, they are encumbered by the intricacies of genetic manipulation, limitations in genome-wide screening, and profound ethical considerations. In contrast, organoids, characterized by their human origins, genomic fidelity, and potential for personalized applications, present a promising frontier, though their implementation is currently impeded by insufficient standardization and substantial costs (Reprinted with permission from Ref. 60. Copyright © 2024 Wiley).

As illustrated in Fig. 3, a comparison of traditional drug screening models reveals critical limitations in established methods. While 2D cell lines afford high-throughput capabilities, their utility is compromised by genomic instability arising from continuous passaging and the absence of a 3D structure, which prevents them from recapitulating the niche-dependent properties of the TME. Conversely, models such as PDX can simulate interactions within the TME, yet they are hampered by interspecies genetic disparities, such as the evolutionary divergence of human tumors within a murine host, as well as the prohibitive costs of whole-genome screening and complex ethical considerations^60^. PDOs, however, surmount these limitations. They faithfully preserve the somatic mutations of the parental tumor; for instance, 90% of somatic mutations found in metastatic CRC biopsies are conserved in their corresponding PDOs. Moreover, they maintain key histopathological features of the primary lesion, including glandular polarity and patterns of stromal invasion^60^. A landmark study, for example, established a library of CRC organoids where the whole-genome sequencing confirmed the preservation of patient-specific *APC/KRAS* mutations and the recapitulation of the original tumor's adenomatous glandular structures. This research also observed that with disease progression, the organoids' dependence on niche factors diminished. These niche factors, exogenous signaling molecules essential for stem cell self-renewal, proliferation, or differentiation, are critical during the initial establishment of organoid cultures. However, with continued evolution, a subset of these organoids acquired the capacity for autonomous growth, becoming independent of external factors. This phenomenon mirrors the evolutionary trajectory observed *in vivo*, where tumors acquire growth independence, thereby validating the high degree of similarity between organoids and their tumors of origin and underscoring their potential as patient “avatars”^61^.

In dual mouse–human models, studies have demonstrated that PDOs can precisely preserve the original tumor's tubular tissue architecture and its characteristic driver mutations, such as *KRAS* and *TP53*. Targeted sequencing analysis of PDOs reveals that their overall mutational profile exhibits an 80%–90% concordance with that of the original tumor^62^. Consequently, serving as a “living biobank” for each patient's tumor^25^, PDOs offer an indispensable platform for both personalized medicine and the study of tumor evolution, delivering a trinity of fidelity, including genetic integrity, histostructural recapitulation, and predictive pharmacological response.

#### 2.1.3 Breakthrough applications of PDOs in drug screening

**Figure 4:**
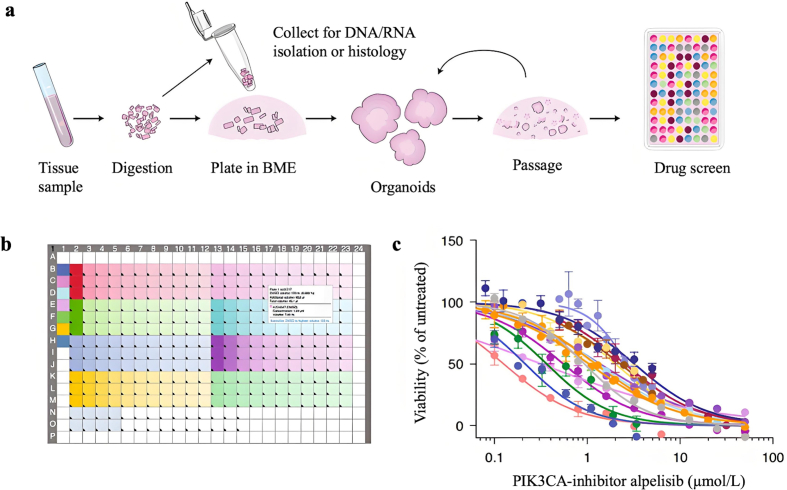
A schematic of the high-throughput drug screening framework for PDOs^56^. Left: (a) A visual representation outlining the procedure for isolating organoids from patient tumors to facilitate drug screening. (b) An illustration of the drug distribution layout within a 384-well cell culture plate, in which distinct colors denote various compounds and the gradations in color intensity correspond to the drug concentration. (c) The cell viability curves for the *PIK3CA* inhibitor, Alpelisib, as tested against head and neck squamous cell carcinoma organoids derived from thirteen unique donors (Adapted with permission from Ref. 56. Copyright © 2020 Springer Nature).

Leveraging their capacity for rapid expansion and amenability to miniaturized culture, organoids have emerged as next-generation platforms for high-throughput drug screening^56^^,^^63^. Their principal advantages lie in their scalability and clinical relevance. A single patient sample, for example, can be expanded through enzymatic digestion and passaging to generate hundreds or even thousands of individual culture units, enabling the parallel testing of numerous drug combinations at varying concentrations in 96- or 384-well plate formats^64^. Furthermore, PDOs faithfully preserve the specific genomic characteristics of the original tumor, ensuring that their drug sensitivity profiles show a strong correlation with clinical outcomes *in vivo*^61^. Key applications include: (1) Accelerating the identification of novel drug targets. During the SARS-CoV-2 pandemic, for instance, a screen of over 1000 FDA-approved drugs using infected pulmonary and colonic organoids identified three potent inhibitors: Imatinib (a viral entry inhibitor), Mycophenolic acid (an RdRp inhibitor), and Quinacrine dihydrochloride (a membrane fusion blocker). This pivotal work propelled Imatinib into a Phase II clinical trial (NCT04394416)^65^ and highlighted the capacity of organoid platforms for rapid response during public health crises. (2) Establishing standardized drug screening platforms. Researchers have developed a standardized protocol for drug screening using head and neck squamous cell carcinoma organoids (Fig. 4), creating a biobank of 13 patient-derived models. These models accurately recapitulate key driver mutations from the primary tumors, such as those in *PIK3CA* and *TP53*, allowing for the quantitative validation of heterogeneous responses to the *PI3KA* inhibitor Alpelisib and enabling its precise alignment with patient-specific genetic profiles^56^^,^^58^. (3) Transforming regulatory paradigms through preclinical validation and clinical prediction. The application of PDOs in drug screening is driving a paradigm shift in regulatory practices. The process began with a comprehensive preclinical validation effort. By screening over 500 bispecific antibodies against a library of more than 500 CRC PDOs, HUB Organoids™ identified MCLA-158, a bispecific antibody targeting *LGR5* and *EGFR*. This antibody was found to selectively eliminate *LGR5+* cancer stem cells while exhibiting markedly lower toxicity in normal colon organoids^62^. In a landmark decision, the FDA approved MCLA-158 for a Phase I/II clinical trial in solid tumors (NCT03526835) based solely on this organoid data. This approval establishes a key precedent, confirming that high-quality organoid data can serve as a sufficient basis for both preclinical validation and clinical prediction to support clinical investigations^66^. (4) Enabling mechanistic integration in drug screening. In studies of pancreatic cancer PDOs, high-throughput screening platforms have been used to map a comprehensive drug sensitivity landscape, revealing a dichotomous response profile to gemcitabine *versus* platinum-based agents. This insight has led to proposed optimizations for therapeutic strategies, thereby advancing the clinical implementation of personalized medicine^59^^,^^67^.

#### 2.1.4 Clinical applications of PDOs

**Figure 5:**
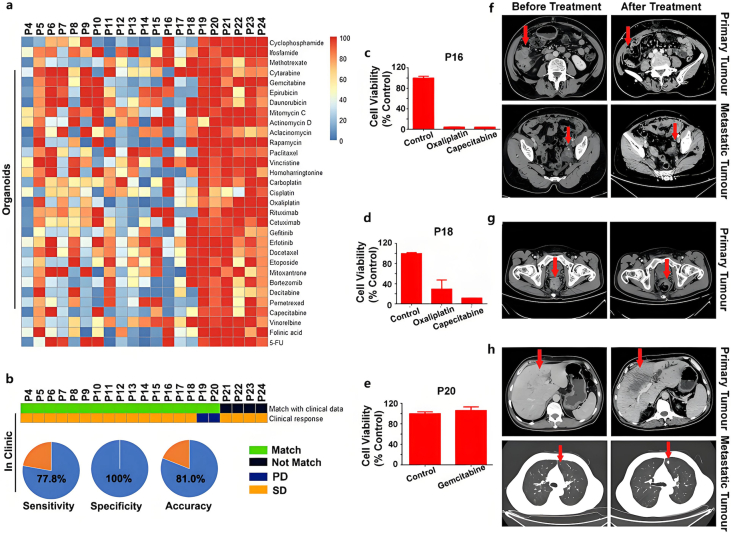
The prediction of patient clinical outcomes utilizing tumor organoids^49^. (a) An organoid viability heatmap illustrating the results from screening tumor organoids, derived from 21 patients, against a panel of 31 anticancer drugs, including 5-fluorouracil. (b) A statistical analysis of the sensitivity, specificity, and accuracy, correlating the outcomes of the organoid drug screen with the clinical therapeutic responses observed in patients. (c–e) Representative pharmacodynamic profiles for patients with cancers of the colon, rectum, and liver, documented before and after treatment. (f–h) Corresponding pre- and post-treatment computed tomography scans for the same patients, which demonstrate a high degree of concordance between the predictions derived from the organoid models and the actual clinical outcomes (Reprinted with permission from Ref. 49. Copyright © 2020 Cell Press (Elsevier)).

The potential of tumor-derived PDOs to predict therapeutic responses represents the cornerstone of their clinical translation. By providing a functional basis for personalized therapy through *ex vivo* drug sensitivity testing, the predictive efficacy of PDOs has been extensively validated across numerous studies. For instance, a landmark study published in Science showed the accuracy of these models for clinical prediction. It demonstrated that screening PDOs from metastatic gastrointestinal cancers against agents like paclitaxel and cetuximab predicted clinical outcomes with 100% sensitivity, 93% specificity, an 88% positive predictive value (PPV), and a 100% negative predictive value (NPV). Crucially, the 100% NPV, a key outcome of this predictive model, strongly suggests that patients whose tumors show drug resistance in organoid models can be spared from undergoing ineffectual treatments^59^. In a real-world study encompassing 107 patients with non-small cell lung cancer, 214 organoid models were established, revealing an overall concordance rate of 83.3% between drug sensitivity assays and clinical therapeutic responses, thus furnishing a critical evidentiary basis for selecting subsequent therapeutic regimens following the development of resistance to *EGFR* tyrosine kinase inhibitors (TKIs)^66^. Another investigation, utilizing a microfluidic platform, successfully compressed the organoid generation timeline to just seven days to facilitate high-throughput screening of 31 anticancer agents for digestive system tumors, including colon, rectal, and liver cancers. This approach achieved a predictive accuracy of 81.0% (100% specificity, 77.8% sensitivity) across 21 patients. The platform's broad applicability across various cancer types, corroborated by clinical efficacy heatmaps (Fig. 5), demonstrated that organoid drug sensitivity results are highly congruent with actual patient outcomes^49^.

Collectively, these studies affirm that organoids serve as robust functional biomarkers capable of accurately predicting clinical responses to drug therapy, with model generation success rates consistently exceeding 85%^46^. Their clinical implementation is poised to significantly advance the establishment of personalized medical decision-making frameworks.

Moreover, PDOs complement the clinical use of next-generation sequencing (NGS) through functional testing. NGS enables high-throughput profiling of exomes, transcriptomes, and whole-genome variants, providing a molecular basis for individualized therapy^68^, ^69^, ^70^. However, only about 33% of patients benefit from NGS-guided treatment; for example, *EGFR* mutations are predictive for targeted therapy but offer limited guidance for chemotherapy and immunotherapy^71^. By directly measuring drug sensitivity, organoid models address the functional interpretation gap in genomics. They can help interpret variants of uncertain significance. In pancreatic cancer organoids, for instance, a subset of *KRAS* wild-type cases showed sensitivity to gemcitabine, which is difficult to predict from genomic data alone^67^. Organoid models can also reproduce multifactorial resistance mechanisms. Their 3D structure can model microenvironment-mediated resistance, including metabolic adaptation and cell–cell interactions, whereas NGS primarily catalogs genetic alterations. In addition, organoids can be used to assess clinical predictive performance. An analysis integrating 17 studies reported that 94.1% of organoid drug sensitivity results correlated with clinical responses, with 29.4% reaching statistical significance and 64.7% showing a clear concordant trend, supporting their potential as predictive biomarkers^68^.

Overall, PDOs preserve the genetic heterogeneity, cellular composition, and microenvironmental features of primary tumors, providing a dynamic functional *in vitro* model. Compared with 2D cell lines and animal models, PDOs are advantageous for studying tumor evolution and guiding individualized drug selection. Nonetheless, several challenges limit clinical implementation, including cancer type-dependent culture success, incomplete representation of the immune microenvironment, and suboptimal cost–effectiveness^13^^,^^60^. For example, the complexity of the stromal milieu contributes to a 68% success rate for breast cancer organoids and 42% for pancreatic cancer organoids^37^. Regarding the immune microenvironment, conventional organoid models face critical hurdles. As they are typically expanded from epithelial stem cells, they lack native immune components. Reconstituting this TME *via* co-culture is hampered by the short lifespan of immune cells, which often lose function within 48–72 h, the drift of cell ratios away from physiological levels, and the absence of an *in situ* antigen presentation environment^37^. Compared with an *in vivo* benchmark of 15%–40%, CD8^+^ T-cell infiltration is below 5% in organoid systems, which constrains immunotherapy evaluation^37^. This deficiency makes it difficult to functionally reproduce the immune checkpoint signaling axis, such as PD1/PDL1, constraining their predictive value for immunotherapy evaluation. The per-sample cost of automated platforms also remains high and requires further optimization^24^. An often overlooked limitation of standard organoid systems is the absence of the host microbiota^72^. This is particularly relevant for gastrointestinal cancers, where the microbiome directly influences carcinogenesis, tumor progression, and therapeutic response to both chemotherapy and immunotherapy^73^. For example, gut microbes like *Fusobacterium nucleatum* have been shown to promote CRC chemoresistance^74^. Reconstituting this complex, often anaerobic, microbial environment alongside aerobic organoid culture presents significant technical hurdles. However, recent advances using microinjection of specific bacterial strains into the organoid lumen or co-culture in specialized organ-on-a-chip platforms are beginning to address this gap, enabling mechanistic studies of host-pathogen interactions, such as the modeling of *Helicobacter pylori*-induced gastric carcinogenesis^75^. Future work must therefore integrate not only immune co-culture but also microbiota to create more holistic tumor models^76^^,^^77^.

### 2.2 Organ-on-a-chip

Organ-on-a-chip systems are microfluidic devices that recapitulate the key functional units of human organs. By integrating living cells with precisely engineered microenvironments, these chips provide a platform to model tissue-level physiology and pathophysiology under dynamic perfusion and mechanical stimulation, which is a major advantage over static culture models, such as organoids^51^.

#### 2.2.1 The development of organ-on-a-chip

Organs-on-chips are generally designed in two categories: barrier models and solid organ models. Barrier model chips are designed to model tissue interfaces and typically feature a two-chamber microfluidic channel separated by a porous membrane. Cells are cultured on one or both sides of the membrane to recreate physiological barriers such as the intestinal epithelium, the lung's alveolar–capillary interface, or the blood–brain barrier^55^^,^^56^. This architecture is ideal for studying nutrient absorption, drug transport, and immune cell trafficking. For solid organ models, to mimic the 3D structure of solid organs such as tumors, organs-on-chips often incorporate 3D culture chambers containing micropillars or microwells. These features serve to confine hydrogel-embedded cells or spheroids, allowing for perfusion of the surrounding 3D tissue construct and enabling studies on metabolism and cell–cell interactions^58^^,^^78^^,^^79^. Recent literature indicates that these organ-on-a-chip systems facilitate the assessment of drug efficacy and safety, particularly for heart, liver, kidney, and tumor models, thereby providing a strategy to reduce reliance on animal testing in preclinical phases^80^^,^^81^.

The fabrication of organ-on-a-chip systems predominantly utilizes soft lithography, photolithography, and additive manufacturing (Table 2)^82^. Soft lithography, often combined with photolithography to generate negative master molds, typically employs elastomeric materials such as polydimethylsiloxane (PDMS). PDMS is characterized by high optical transparency (240–1100 nm), gas permeability, and biocompatibility. However, hydrophobicity and non-specific adsorption of small molecules remain significant limitations for drug screening assays. Thermoplastic polymers, such as polymethyl methacrylate (PMMA) and polystyrene (PS), processed *via* hot embossing or injection molding, are used when rigid, low-adsorption substrates are required^79^^,^^83^.

**Table 2:** A comparative overview of fabrication strategies for organ-on-a-chip systems^82^.

Fabrication strategy	Core mechanism/materials	Key advantages	Primary limitations
Soft lithography	Replica molding using elastomeric polymers, such as polydimethylsiloxane (PDMS), on a master mold.	High optical transparency (240–1100 nm). Gas permeability. Rapid prototyping.	Hydrophobic surface. Non-specific adsorption of small hydrophobic molecules. Requires master mold fabrication.
Photolithography	Light exposure through a mask to pattern photoresist on silicon wafers.	High resolution (<10 μm) Precise control of micro-features.	High cost. Complex cleanroom procedures. Typically limited to 2D or 2.5D structures (master molds).
Stereolithography (SLA)	Layer-by-layer photopolymerization of liquid resin using a laser.	High precision. Smooth surface finish suitable for micro-channels. Capable of complex internal geometries.	Resin toxicity requires rigorous post-processing for biocompatibility. Material choices are limited compared to FDM.
Selective laser sintering (SLS)	Laser sintering of powdered materials (nylon, polyamide, metals).	Robust structural integrity: no support structures needed. Wide range of material compatibility.	Rougher surface finish (porous) may affect fluid flow. High processing temperatures.
Fused deposition modeling (FDM)	Extrusion of heated thermoplastic filaments, such as polylactic acid and acrylonitrile butadiene styrene.	Cost-effective. Simple operation. Uses biocompatible thermoplastics.	Lower resolution compared to SLA. Risk of leakage between layers. Rough surface finish.
3D bioprinting	Deposition of bioinks containing living cells and hydrogels.	Simultaneous fabrication of scaffold and tissue. Precise spatial distribution of multiple cell types.	Mechanical stability of hydrogels is lower than that of polymers. Complex optimization of cell viability during printing.

Additive manufacturing, particularly 3D printing, has emerged as a viable strategy for fabricating monolithic devices with complex 3D geometries not attainable *via* traditional molding^82^. Among the key techniques, stereolithography (SLA) utilizes laser-mediated selective curing of liquid resins; due to its high resolution and superior surface quality, it is particularly applicable for fabricating micro-channels and valves requiring precise fluidic regulation. Selective laser sintering (SLS) offers a distinct approach by sintering powdered polymers or metals to construct durable base structures and interconnected fluidic networks, offering compatibility with a broader range of materials. For more cost-sensitive applications, fused deposition modeling (FDM) provides a solution based on the extrusion of thermoplastic filaments, serving as an effective method for fabricating device frames, albeit with lower resolution compared to SLA^82^. Extending beyond these structural methods, 3D bioprinting deposits living cells and bioinks to construct functional tissue scaffolds. Unlike acellular printing, this technique facilitates the precise spatial organization of heterogeneous cell populations, enabling the direct generation of vascularized or multi-layered tissue constructs within microfluidic platforms^82^^,^^84^.

**Figure 6:**
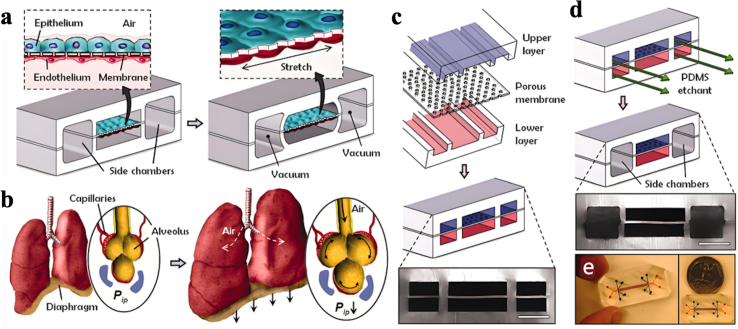
The structure of the first lung-on-a-chip and its mechanism for simulating the mechanics of breathing^79^. (a) Biomimetic alveolar-capillary barrier chip design: This microfabricated lung model features a compartmentalized polydimethylsiloxane (PDMS) microchannel structure. It reconstructs the alveolar-capillary barrier on a thin, porous, and flexible PDMS membrane coated with ECM. By applying a vacuum to the side chambers, the device mimics physiological breathing movements, which induce mechanical stretching of the PDMS membrane at the alveolar-capillary interface. (b) Principle of physiological breathing simulation: During *in vivo* inspiration, the contraction of the diaphragm lowers intrapleural pressure. This leads to the expansion of the alveoli, thereby stretching the alveolar-capillary interface. (c) Key microfabrication technique: Three layers of PDMS are aligned and irreversibly bonded to form two main channels separated by a 10 μm thick PDMS membrane. This membrane is perforated with an array of through-holes, each with an effective diameter of approximately 10 μm (Scale bar: 200 μm). (d) Post-bonding processing: A PDMS etchant is introduced into the side channels to selectively etch away portions of the membrane, creating two large side chambers. Applying a vacuum to these chambers facilitates the mechanical stretching of the membrane (Scale bar: 200 μm). (e) Top-down photograph of the fabricated lung-on-a-chip microfluidic device (Adapted with permission from Ref. 79. Copyright © 2010 American Association for the Advancement of Science).

Since the pioneering creation of the first alveolar–capillary barrier chip in 2010 by the team of Donald E. Ingber (Fig. 6), which successfully simulated respiratory mechanics and validated transmembrane gas exchange, the field has undergone three major stages of technological advancement (Table 3)^78^^,^^79^^,^^85^^,^^86^.

**Table 3:** Technological milestones in the development of organ-on-a-chip.

Phase	Pivotal advances	Scientific significance
Single-organ modeling (2010–2015)	Pulmonary edema-on-a-chip model: Reconstituted the pathophysiology of vascular leakage and led to the discovery of the endothelial barrier-protective agent, GSK2193874^78^^,^^79^^,^^85^.	Provided the first demonstration of an organ-on-a-chip's integrated capacity for disease modeling and drug development.
Multi-organ interaction (2015–2020)	Multi-organ-on-a-chip models (intestine, liver, skin, kidney): Sustained functional coupling for 28 days, enabling the *in vitro* simulation of *in vivo* ADME (absorption, distribution, metabolism, and excretion) processes^87^. The AngioChip™: a Platform for engineering perfusable, three-dimensional microvascular networks with vascularization efficiencies exceeding 85%^88^.	Addressed the challenge of sustaining long-term, functional multi-tissue interactions *ex vivo*.
Automation and clinical translation (2020–Present)	The interrogator system: An automated platform for the co-culture and *in situ* monitoring of eight distinct vascularized organ-on-a-chip models^89^. Patient-derived multi-organ chips: Modeled communication between cardiac, skeletal, hepatic, and dermal tissues, mediated by circulating immune cells^90^.	Advanced personalized treatment studies toward enhanced standardization and high-throughput capabilities.

##### 2.2.1.1 Core advantages of organ-on-a-chip systems

**Figure 7:**
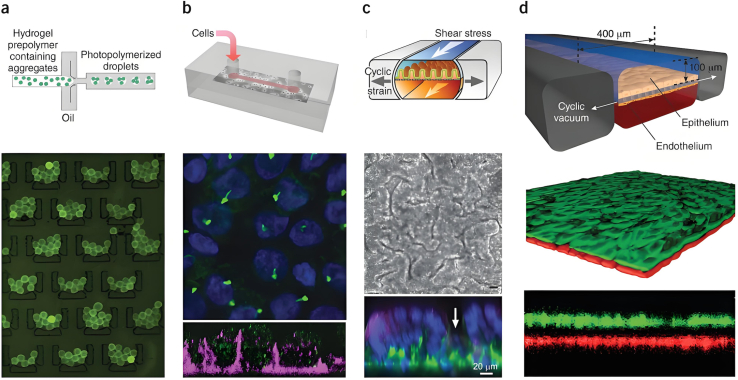
Four representative organ-on-a-chip devices. (a) A liver-on-a-chip, featuring a dual-layer structure with upper and lower microfluidic channels separated by a PET membrane, complete with an inlet and an outlet (Reprinted with permission from Ref. 91. Copyright © 2018 Royal Society of Chemistry). (b) A kidney-on-a-chip, composed of renal proximal tubule epithelial cells (top). The bottom panel is a fluorescence micrograph demonstrating the functional expression of the Na^+^/K^+^ ATPase protein. (c) An intestine-on-a-chip, where human intestinal epithelial cells are cultured on an ECM-coated membrane. Peristalsis-like motions are simulated by applying cyclic suction to the side chambers (top). Below, bright-field and fluorescence micrographs illustrate the formation of intestinal crypt-like structures. (d) A lung-on-a-chip, with human alveolar epithelial cells and vascular endothelial cells co-cultured on the upper and lower sides of a PDMS membrane, respectively. Respiratory movements are simulated *via* cyclic suction applied to side channels (top). The cross-sectional view below demonstrates that the alveolar epithelium (green) and the vascular endothelium (red) successfully reconstitute the alveolar–capillary interface (bottom) (Reprinted with permission from Ref. 78. Copyright © 2014 Springer Nature).

The central technological advantage of organ-on-a-chip platforms lies in their integrated control of mechanical forces, such as shear stress and cyclic stretch, biochemical gradients, and cell–cell interactions *via* microfluidic perfusion chambers and porous-membrane architectures (Fig. 7), thereby dynamically reconstructing tissue microenvironments close to physiological states ^34^^,^^91^. For example, a liver-on-chip with embedded biosensors enables real-time dual monitoring of vascularization and hepatic function. This microfluidic system showed that fluid mechanical stimulation synchronously promotes the co-maturation of endothelial cells and hepatocytes. 3D electrochemical sensors detected a critical inflection point on day 5, aligning with the theoretically predicted transplantation window. Transplantation within this window led to 93% regression of fibrosis and lobular reconstruction in a cirrhotic mouse model, whereas transplantation before or after the window reduced efficacy by 50%–70%^91^. These findings validate the transplantation window hypothesis and provide a quantitative framework for quality control in organoid-based regenerative therapies^92^. A multi-chamber lung-on-chip recapitulates tissue interfaces and applies 10%–15% cyclic strain to mimic breathing, increasing epithelial sodium channel (ENaC) expression by 3.2-fold and accurately modeling gas exchange and immune responses across the alveolar–capillary barrier^91^. As detailed previously^93^, microfluidic platforms that mimic the liver sinusoid architecture have been instrumental in maintaining long-term hepatocyte viability and function. By providing dynamic perfusion and precise control over biophysical cues like shear stress, these systems significantly outperform static cultures. They have been pivotal not only for *in vitro* applications like disease modeling and drug toxicity testing but also as core components in developing next-generation bioartificial liver (BAL) devices. These BAL systems aim to provide extracorporeal support for patients with acute liver failure, bridging the gap to transplantation^93^.

At the level of inter-organ crosstalk, porous-membrane co-culture, as in gut-liver chips, reproduces the first-pass effect with a bioavailability prediction error of less than 15%; microfluidically linked multi-organ systems, such as heart–liver–kidney chips, enable a 28-day functional simulation of the full ADME process^78^^,^^89^. In particular, the incorporation of circulating immune cells allows interrogation of inter-organ signaling and elucidation of pathologies such as cytokine storms^87^. This highly biomimetic milieu has driven tangible translational gains. In drug development, the vascular leak inhibitor GSK2193874, developed using a pulmonary edema model, achieved a predicted inhibition rate of 92% (*versus* 85% in animal models). Several multi-organ chips have increased preclinical toxicity prediction accuracy to 89%, compared with 70% for traditional models^34^^,^^94^, providing a new platform to guide precision pharmacotherapy^87^.

##### 2.2.1.2 Organ-on-a-chip for disease modeling and drug response prediction

Organ-on-a-chip platforms excel at recapitulating TME specificity and accurately predicting drug responses^34^^,^^91^. Their power lies in reconstructing organ-specific cancer behaviors that are often lost in conventional models. For example, by injecting non-small cell lung carcinoma (NSCLC) cells into primary alveolar and small-airway chips, Donald E. Ingber's group demonstrated that cancer cells proliferated more robustly in the alveolar chip, faithfully mirroring the clinical preference of NSCLC for growth in alveolar regions^34^. However, modeling the full complexity of cancer requires more than just organ specificity; it demands a faithful reconstruction of the TME. The TME is a central engine of tumor progression, driving therapeutic resistance through physical barriers, signaling crosstalk, and immune suppression^85^^,^^95^. Similarly, a stiffness-tunable cholangiocarcinoma chip demonstrated that physiological matrix stiffening significantly enhances chemoresistance and promotes epithelial–mesenchymal transition^96^. While the aforementioned lung cancer model captured proliferative behavior, it was limited in its reconstruction of the stromal compartment. To address this, a biomimetic liver tumor chip was developed by integrating decellularized liver matrix (DLM) with gelatin methacryloyl (GelMA). This platform precisely tuned matrix stiffness and fluid shear, markedly enhancing microenvironmental fidelity. Compared to standard GelMA-only chips, hepatocellular carcinoma cells in the DLM-GelMA environment exhibited higher viability, increased functional protein expression, and, critically, altered drug responses. While their response to sorafenib was consistent with prior reports, they showed significantly heightened sensitivity to acetaminophen, highlighting how a biomimetic TME can modulate drug pharmacokinetics^85^. A key aspect is that the dynamic microenvironment of organ-on-a-chip systems makes them suitable for modeling cancer hallmarks that depend on mechanical or transport processes. For instance, they can simulate activating invasion and metastasis by applying fluidic shear stress, a stimulus that can induce a >3-fold increase in cancer cell invasion compared to static conditions. These platforms are equally applicable to modelling inducing angiogenesis, where they can achieve over 85% vascularization efficiency in engineered microvascular networks^88^, providing a basis for dissecting mechanisms that static models cannot capture.

##### 2.2.1.3 Organs-on-chips catalyze a paradigm shift in drug development

Organs-on-chips are now deeply integrated across the drug development continuum, for example: (1) Accelerating neurotherapeutic discovery. A neural chip integrating microelectrode arrays with microfluidics reconstructed an autoimmune demyelination model and identified TNT005, a monoclonal antibody that restores synaptic transmission; the clinical trial advanced on the basis of chip data (NCT04658472) has received FDA Breakthrough Therapy designation^15^^,^^97^. (2) High-throughput antiviral screening. The Donald E. Ingber team established a SARS-CoV-2 infection model in a dual-channel human airway chip and discovered that amodiaquine, toremifene, and clomiphene markedly inhibit viral replication, prompting two clinical trials (NCT04532931, NCT04502342)^98^. (3) Optimizing formulations for metastatic cancer therapy. By combining organ-on-chip platforms with organ-specific decellularized extracellular matrix (dECM), investigators built a 3D metastasis-on-chip to model renal cell carcinoma spread to the liver and to predict therapeutic responses. Varying the proportions of Caki-1 and HepLL cells to emulate different stages of hepatic metastasis revealed a linear inverse correlation between the efficacy of 5-fluorouracil and the fraction of Caki-1 cells. Moreover, compared with free 5-fluorouracil, PLGA–PEG nanoparticles loaded with 5-fluorouracil (PLGA-PEG/5-FU NPs) produced stronger cytotoxicity at equivalent concentrations^99^. Relevant studies further indicate that microfluidic technologies enable the precise synthesis of lipid and polymer nanocarriers with controllable physicochemical properties, optimizing their application in drug delivery systems^81^.

Taken together, organs-on-chips not only accelerate high-throughput screening and functional validation of conventional chemotherapeutics, including small-molecule inhibitors and antibody drugs, but also provide indispensable advantages for evaluating emerging therapeutic strategies. However, widespread adoption of organ-on-a-chip is hampered by critical challenges, including a lack of standardization, low throughput, and, most notably, an oversimplified biology that can limit clinical correlation^99^^,^^100^. To specifically address this biological fidelity gap, organoid-on-a-chip represents a significant leap forward. This approach seeds high-fidelity organoids into microfluidic platforms and introduces physiological cues, such as perfusion and mechanical forces, thereby creating models with high biological relevance and predictive power, as extensively reviewed for applications in personalized medicine^27^^,^^101^.

#### 2.2.2 Organoid-on-a-chip: integrating strengths to reshape *in vitro* models

Organoid-on-a-chip platforms represent a frontier of interdisciplinary bioengineering, constructed by integrating 3D organoids, which self-organize from stem cells, with microfluidic organoids-on-a-chip platforms^27^^,^^100^. The essence of this system lies in harnessing the inherent capacity of stem cells for multi-lineage differentiation and spontaneous spatial organization. Within a microenvironment precisely modulated by the microfluidic system, encompassing parameters such as fluid shear stress, dissolved oxygen gradients, and the concentration of biochemical signaling molecules, these platforms facilitate the *in vitro* reconstruction of functional organ units with high physiological relevance. In contrast to conventional organ-on-a-chip strategies that rely on the artificial assembly of cell populations, organoids-on-a-chip can generate cellular heterogeneity and complex tissue architecture that more faithfully recapitulates that of native organs^97^^,^^102^. The field has witnessed a series of significant breakthroughs in recent years, with its core technological innovations categorized as follows (Table 4)^97^^,^^102^, ^103^, ^104^, ^105^.

**Table 4:** Summary of breakthroughs and progress in organoids-on-a-chip.

Year	Core technological innovation	Technical advantages	Key breakthroughs and application contributions	Field of contribution
2019	Recreating organ-specific functions	High-fidelity modeling of tumor structures	Reconstructed functional renal tubules and their transepithelial transport capabilities on-chips.	Mechanistic studies of genetic diseases
		Personalized model platform	Mechanistic studies of genetic diseases	Precision medicine^103^
2019	Microfluidic regulation of tissue maturation	Precise control of the microenvironment	Revealed the key role of fluidic shear stress in the maturation of vascular networks in kidney organoids.	Optimization of tissue engineering^104^
2020	Automated high-throughput drug screening and dynamic dosing	High-throughput platform	Identified inter-patient differences in drug response among pancreatic cancer patients.	Precision oncology
		Personalized treatment strategies	Demonstrated that dynamic dosing (*e*.*g*., scheduled dose adjustments) is superior to conventional constant-dose therapy.	Optimization of clinical drug administratio^105^
2022	Multi-organ partitioned co-culture system	Integration of multi-organ interactions	A liver–islet organoid co-culture system simulated glucose metabolic responses, advancing beyond static models.	Metabolic disease research
		Maintenance of long-term homeostasis	Overcomes limitations of static models	Mechanistic studies of chronic diseases^106^
2022	Personalized precision therapy	Disease modeling and drug screening for rare diseases	An electrophysiological model for (Chronic Inflammatory Demyelinating Polyneuropathy, CIDP) and (Multifocal Motor Neuropathy, MMN) recapitulated clinical neurophysiological features. This model supported an investigational new Drug (IND) application.	Pathological mechanism research and new drug development for rare diseases^105^
2024	Environmental pathology modeling platform	Complex pathology modelling	Established a model of chronic cardiac injury induced by microplastics.	Environmental health research^97^^,^^102^
		Analysis of emerging risk factors	Provided an *in vitro* paradigm for studying the pathogenic mechanisms of pollutants.	Support for public health policy

These findings demonstrate that organoids-on-a-chip combines the advantages of both organoids and organ-on-a-chip systems, driving breakthroughs in multiple fields. Its capacity for high-fidelity organ simulation, based on stem cell self-organization and precise microenvironmental control, has significantly deepened our understanding of disease mechanisms and promoted the replacement of animal testing. The ability to model dynamic multi-organ interactions, which relies on integrated multi-chamber microfluidic designs, provides crucial support for evaluating systemic drug toxicity and predicting treatment outcomes. Automated, high-throughput platforms, through the coordinated integration of sensors and fluid control systems, greatly accelerate the generation of individualized treatment strategies and enhance the efficiency of precision dosing decisions. The technology to reconstruct pathological microenvironments, using controlled delivery of disease-related factors, empowers research in environmental medicine and helps establish safety thresholds for toxic substances. Furthermore, patient-specific modeling using PDO systems facilitates a shift toward a data-driven paradigm, enabling the prediction of clinical responses to treatment^27^^,^^100^^,^^102^. Collectively, these breakthroughs establish a complete value chain from basic research to clinical application, offering innovative tools for advancing precision medicine and informing risk assessment.

##### 2.2.2.1 Applications of organoids-on-a-chip in personalized drug testing

**Figure 8:**
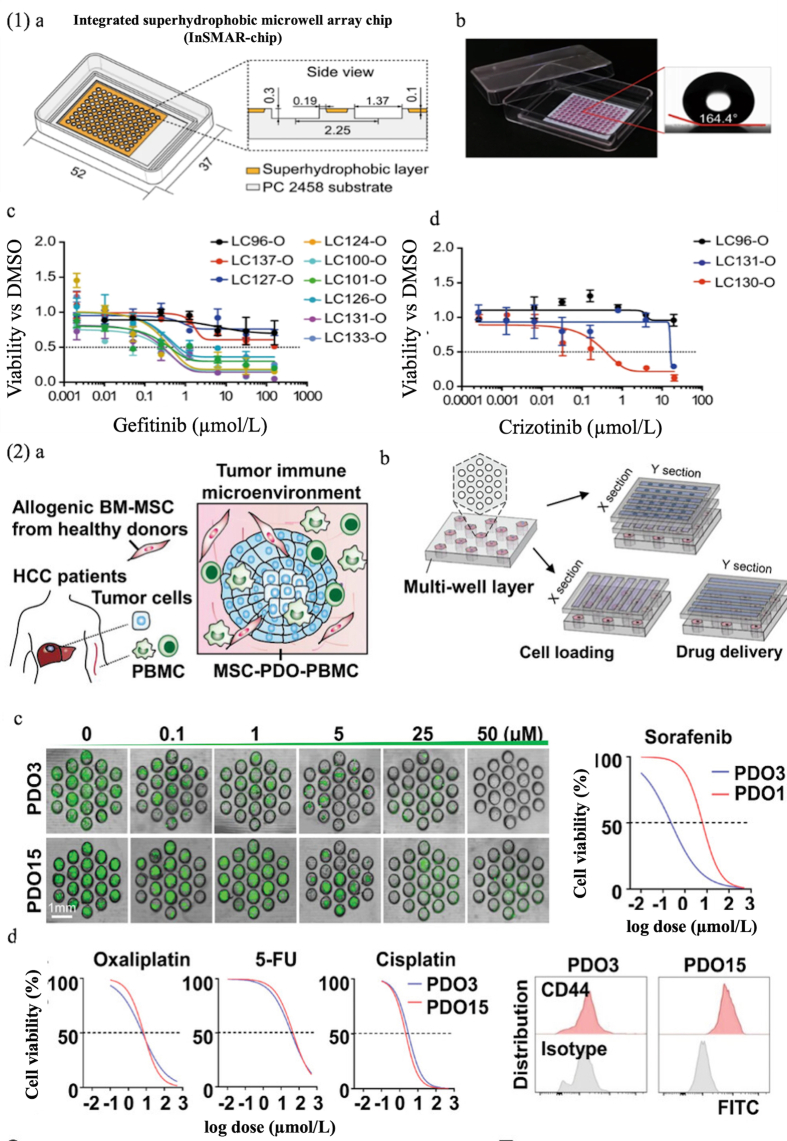
An organoid-on-a-chip platform for assessing drug sensitivity in cancers. (1a) Schematic illustration (left) and cross-sectional view (right) of the organoids-on-a-chip. The integrated superhydrophobic microwell array (InSMAR) chip, with dimensions of 52 × 37 mm^2^, is manufactured from polycarbonate 2458 *via* a standard injection molding process. Its surface incorporates an array of 108 microwells (1.37 mm diameter, 300 μm depth, 2.25 mm pitch), each with an approximate volume of 440 nL. (1b) The microwell region features a 100 μm deep recessed top surface, which is coated with a superhydrophobic layer. (1c) The response of lung cancer organoids (LCOs) to the TKI inhibitor gefitinib correlates with their *EGFR* mutation status. The fitted dose–response curves illustrate the viability of nine LCO lines across a gradient of gefitinib concentrations. Three of these lines are *EGFR* wild type, while the remaining six harbor *EGFR* activating mutations known to confer sensitivity to TKI inhibition. (1d) Response of LCOs to crizotinib, an anaplastic lymphoma kinase (ALK) inhibitor. The organoid line harboring the *EML4-ALK* rearrangement (LC130-O) exhibits significantly diminished viability, whereas the *ALK* wild-type organoids (LC96-O and LC131-O) are non-responsive (Adapted with permission from Ref. 107. Copyright © 2021 Springer Nature). (2a) Schematic of the hepatocellular carcinoma TME model. The model integrates PDOs with autologous peripheral blood mononuclear cells and allogeneic bone marrow-derived mesenchymal stem cells to recapitulate the TME. (2b) Design of the high-throughput microfluidic chip. The three-layer device consists of orthogonal channels for cell and drug delivery and a bottom layer containing 36 microwell arrays for parallel organoid culture and screening. (2c) Chemosensitivity assessment of TME models to Sorafenib. Representative fluorescence images of Calcein AM-stained PDO3 and PDO15 after 6 days of treatment, and their corresponding dose–response curves. (2d) Drug response profiling and biomarker analysis. Dose–response curves of PDO3 and PDO15 models for Oxaliplatin, 5-FU, and Cisplatin. Flow cytometry analysis shows the differential expression of CD44 in the two PDO lines (Adapted with permission from Ref. 108. Copyright © 2023 Wiley-VCH).

Organoids-on-a-chip, by dynamically recapitulating patient-specific TME, offers a critical technological platform for personalized drug testing. Its core value is demonstrated through several key aspects:(1) High-fidelity simulation of the TME. By culturing organoids derived from clinical lung cancer specimens within a perfused microfluidic chip**,** studies demonstrated that the model could overcome the nutrient limitations of static cultures. This dynamic environment not only enabled the organoids to faithfully retain the histological architecture and genetic profile of the primary tumor for extended periods but also recapitulated key aspects of the native TME, thereby establishing a robust and more physiologically relevant foundation for drug sensitivity assays (Fig. 8)^107^. Numerous such platforms have been cataloged in recent reviews. A key finding is that by combining PDOs with microfluidic control, these systems can achieve high concordance with clinical responses. For instance, in lung and pancreatic adenocarcinoma, this concordance often exceeds 80%–90% for both targeted therapies and chemotherapies^101^. Moreover, a pancreatic ductal adenocarcinoma (PDAC) chip model, by co-culturing pancreatic stellate cells and macrophages, successfully emulated the *in vivo* pharmacodynamic response to the chemotherapeutic agent gemcitabine, validating organoids-on-a-chip's capacity to reconstruct complex TME and elucidate drug mechanisms of action^100^. (2) Achieving clinical prediction through preclinical validation. On the aforementioned lung cancer organoids-on-a-chip platform, the results from sensitivity testing for drugs such as gefitinib and crizotinib exhibited a perfect (100%) correlation in both accuracy and specificity with the clinical outcomes observed in a cohort of ten patients^107^. Furthermore, a hepatocellular carcinoma organoids-on-a-chip, developed by integrating a co-culture system of mesenchymal stromal cells and peripheral blood mononuclear cells, effectively simulated the TME while enhancing the efficiency and uniformity of organoid cultures^108^^,^^109^. This approach substantially abbreviated the timeline for high-throughput organoid culture and drug screening, markedly improving the predictive efficacy for clinical responses to treatments such as immune checkpoint inhibitors (ICIs)^108^. Similarly, biomimetic liver cancer-on-a-chip models have been employed to elucidate tumor–stroma interactions, identifying Lipocalin 2 (LCN2) as a factor contributing to tumor progression and sorafenib resistance^110^.

Collectively, these findings demonstrate that tumor organoids-on-a-chip platforms enable rapid and precise prediction of therapeutic responses by creating a high-fidelity, dynamic patient-specific TME^100^. By integrating perfusable micro-channels and segregated co-culture compartments**,** this technology uniquely allows for the recreation of an active immune microenvironment and the real-time analysis of cellular interactions and dynamic biomarkers^111^. This ability to capture complex, time-resolved mechanisms of action for novel therapeutics^100^ not only accelerates clinical decision-making but also solidifies the pivotal role of organoids-on-a-chip in driving the paradigm shift towards a microenvironment-focused precision medicine framework^111^.

##### 2.2.2.2 Innovative applications of organoids-on-a-chip for predicting off-target drug toxicity

**Figure 9:**
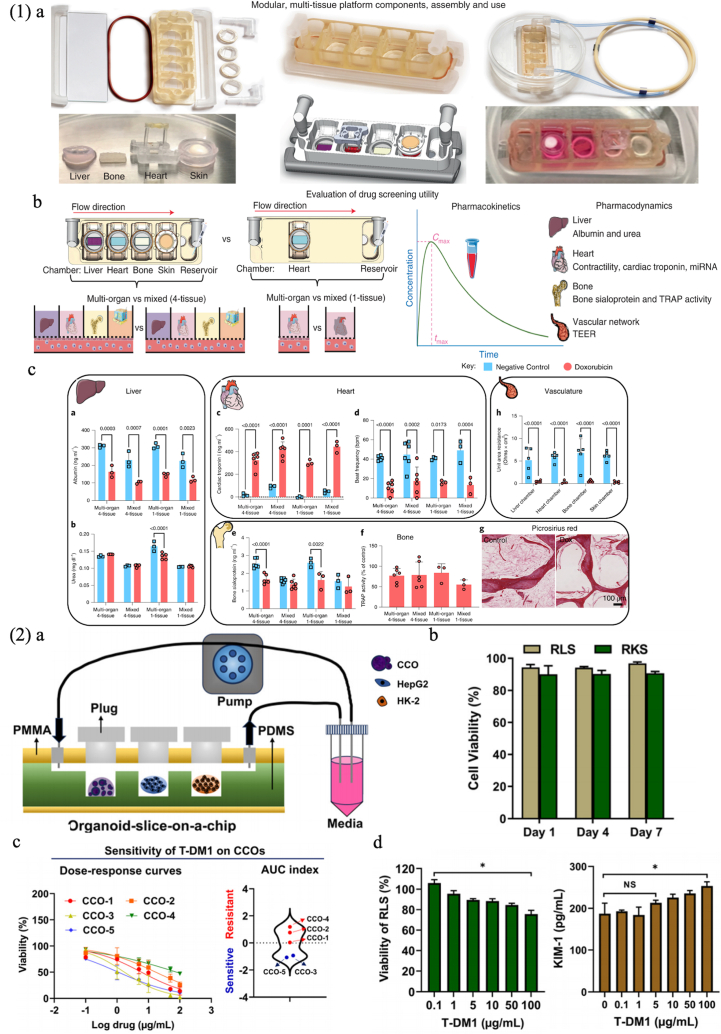
Multi-organ-on-a-chip platforms for drug screening. (1a) Design and assembly of the modular multi-organ chip. Top row: Photographs illustrating the “plug-and-play” system, featuring individual organ chambers and an underlying common vascular channel. Bottom row: Tissues shown before and after being loaded into the device, with distinct media colors demonstrating the fluidic isolation maintained for each organ compartment. (1b) Experimental design for doxorubicin screening. The schematic illustrates the direct comparison of pharmacokinetic (PK) and pharmacodynamic (PD) responses between the multi-organ chip and a mixed-tissue control chip, tested under both multi-tissue and single-tissue culture configurations. (1c) Organ-specific functional characterization after 72 h. Key functional readouts include Liver: albumin and urea secretion. Cardiac: cardiac troponin secretion and contractility. Bone: secreted bone sialoprotein, TRAP activity, and representative immunohistochemistry (IHC). (*n* = 3–6 biological replicates; scale bar, 100 μm) (Adapted with permission from Ref. 90. Copyright © 2022 Springer Nature). (2) A multi-organ-on-a-chip for studying drug efficacy and toxicity. (2a) Schematic of the multi-organ-on-a-chip integrating cholangiocarcinoma organoids (CCOs), rat liver spheroids (RLS), and rat kidney spheroids (RKS). For anticancer drug assessment, RLS, RKS, and CCOs were seeded into the culture wells, which were subsequently sealed with plugs. (2b) Viability of CCOs, RLS, and RKS after 7 days of co-culture in the perfusion chip. (2c) Drug response curves and sensitivity to T-DM1 exhibited by CCOs derived from different patients. (2d) Analysis of RLS viability on Days 1, 4, and 7, and measurements of secreted kidney injury molecule-1 (KIM-1) under various drug concentrations (Reprinted with permission from Ref. 112. Copyright © 2025 Royal Society of Chemistry).

Conventional drug testing often prioritizes on-target efficacy while overlooking off-target toxicity and unintended injury to non-target organs, which contributes to roughly 30% of clinical drug development failures due to unpredictable toxic reactions^34^^,^^91^. To address the disjunction between efficacy and toxicity assessment in oncology, a microfluidic multi-organ chip was developed (Fig. 9)^112^. Through dynamic perfusion, the device functionally links cholangiocarcinoma organoids (CCOs), recellularized liver slices (RLS), and recellularized kidney slices (RKS) in series, forming a highly biomimetic liver–kidney–tumor interaction model. Over 7 days of perfusion culture, all tissues maintained robust viability, and the dynamic microenvironment markedly enhanced organ function: RLS exhibited significantly higher urea and albumin secretion than under static culture, while RKS showed more than a twofold increase in albumin and glucose uptake, indicating augmented hepatic secretory activity and renal reabsorptive capacity. In tests with the antibody–drug conjugate T-DM1, the platform concurrently yielded two critical readouts. For antitumor efficacy, T-DM1 exerted pronounced inhibitory effects on CCOs, with substantial inter-individual variability in sensitivity. To stringently assess off-target effects, healthy liver (RLS) and kidney (RKS) spheroids were exposed to a high concentration of 100 μg/mL. Even under this condition, cell viability remained substantial at 75.67 ± 3.64% and 81.03 ± 5.69%, respectively. These results suggest a wide therapeutic window with modest hepato- and nephrotoxicity, aligning with the high precision expected from an antibody–drug conjugate platform^112^. This approach enables dual evaluation on a single platform, shortening efficacy and toxicity assessment timelines, quantifying the impact of organ crosstalk on toxicity thresholds, and providing hepatic-renal toxicity alerts for individualized dosing, of particular value for patients with baseline liver or kidney dysfunction.

##### 2.2.2.3 Organ/organoid-on-a-chip technologies

Organ-on-a-chip and organoid-on-a-chip technologies are fundamentally reshaping the paradigm of preclinical drug development, a shift supported by growing regulatory acceptance. In 2022, the FDA, for the first time, approved an IND application for Sanofi's antibody therapeutic, TNT005, based on data from an organ-on-a-chip platform, signifying the technology's formal endorsement^15^. This tipping point is underpinned by robust scientific validation. For instance, a liver-chip system from Emulate, Inc., featuring a biomimetic dual-channel architecture, was tested in a large-scale study of 27 drugs across 870 chips. The platform demonstrated exceptional predictive power: it correctly identified 87% of clinically hepatotoxic drugs that had been missed in animal testing, while maintaining 100% specificity for safe compounds. It is estimated that the widespread adoption of this technology could enhance annual research and development productivity across the industry by $3 billion^113^.

This technological maturation is driving a global wave of industrial translation. European pioneers, such as Mimetas and CN-Bio, are seeing their platforms adopted by major pharmaceutical companies^13^. In China, academic-industry consortia are building multi-organ and tumor-immune chip programs to support IND-enabling studies and early clinical decision-making, and although many activities are documented mainly through company communications rather than peer-reviewed publications, the trend points to wider preclinical qualification and increasing use alongside conventional assays^13^.

At the current frontier, these platforms are evolving to capture even greater biological complexity. Tumor organoid-on-a-chip systems, integrating PDOs, now reconstruct the *in situ* immune microenvironment to model tumor heterogeneity and therapeutic response with high fidelity. The convergence of these technologies is seen in interconnected multi-organ systems, which functionally link discrete organ-on-a-chip platforms *via* microfluidic channels. These systems, often termed body-on-a-chip or multi-organ-on-a-chip (MOOC), are designed to recapitulate systemic inter-organ crosstalk, an aspect of human physiology that single-organ models cannot capture. This capability is critical for studying metastatic processes and systemic drug responses, as it allows for the mimicry of dynamic interactions between primary and secondary tumor sites and the elucidation of organ-specific toxicities^89^^,^^114^^,^^115^.

In the context of cancer metastasis, MOOCs provide a platform to investigate the mechanisms of organotropism and the metastatic cascade. By circulating fluid between a primary tumor chamber and a downstream target organ chamber, these systems model key steps including intravasation, circulation, and extravasation. For instance, a metastasis-on-a-chip was developed to model the progression of renal cell carcinoma to the liver by co-culturing cancer cells and hepatocytes. This system simulated colonization at the secondary site and revealed a linear negative correlation between the efficacy of 5-FU and metastatic progression. Quantitative data from this platform also demonstrated that 5-FU loaded into PLGA-PEG nanoparticles induced significantly higher cytotoxicity than the free drug at equivalent concentrations, validating its utility for optimizing formulations for metastatic disease^99^.

For modeling systemic drug responses and organ-specific toxicity, interconnected systems offer a holistic assessment of pharmacokinetics and pharmacodynamics (PK/PD). Integrated multi-organ platforms allow for the simultaneous evaluation of on-target anti-tumor efficacy and off-target damage. In one such study, a microfluidic circuit tandemly linked cholangiocarcinoma organoids with decellularized liver and kidney slices to model the systemic effects of the antibody–drug conjugate T-DM1. The platform provided precise toxicity data, showing that while T-DM1 effectively inhibited tumor growth, the viability of non-targeted liver and kidney tissues remained at 75.67 ± 3.64% and 81.03 ± 5.69%, respectively, at a concentration of 100 μg/mL^112^. In another advancement, a system linking cardiac, liver, bone, and skin tissues achieved functional coupling for up to four weeks. This platform enabled the study of doxorubicin metabolism, where the presence of the liver module altered the drug's cardiotoxicity profile compared to single-organ controls (Fig. 9)^90^. These interconnected systems overcome the fragmentation of traditional models and provide a quantitative basis for predicting systemic interactions and therapeutic windows.

The ambition of this field, however, extends even beyond preclinical modeling, aiming to create functional therapeutic systems. A compelling example is the development of a bioartificial liver support system (BLSS). This system integrates a bioengineered whole liver, constructed from a DLM and GelMA hydrogel, within an oxygenated bioreactor. Beyond preclinical modeling, enabling technologies continue to improve platform scalability. A microfluidic self-perfusion chip (MSPC) that enables pumpless 3D culture further supports scalable maintenance of 3D cell, microtissue, and organoid constructs^116^.

### 2.3 Microtumors

Microtumors, also known as tumoroids or patient-derived tumor-like cell clusters (PTCs), are 3D *ex vivo* models that distinctly preserve not only the cancer cells but also key components of the native TME, including stromal cells and tumor-infiltrating immune cells^117^. This TME preservation is their defining advantage over traditional PDOs or cancer cell lines, making them an emerging powerful tool for immuno-oncology research and personalized drug screening^118^. Two main methodological strategies are used for their generation.

#### 2.3.1 Methodological approaches to microtumor generation

Microtumors represent a class of advanced *in vitro* 3D models engineered directly from a patient's autologous tumor tissue. Their defining characteristic is the ability to preserve, at a micro-scale, the tumor's native 3D architecture and complex microenvironment, including the intricate network of tumor cells, stromal components, and immune infiltrates. This high degree of biological fidelity positions them as powerful tools for precision oncology, bridging the gap between simplified models and clinical reality. Two principal strategies have emerged for constructing these high-fidelity microtumor models: a “top-down” approach using tissue fragments and a “bottom-up” approach based on cellular self-assembly^117^.

The “top-down” strategy uses tumor slices to preserve native tissue architecture, but their fragility under perfusion has historically limited their lifespan. Bioengineering is now overcoming this challenge. For instance, a study engineered a “tumor slice sandwich” chip that encapsulates a patient-derived slice within a protective, biomimetic hydrogel (DLM-GelMA). This design shields the tissue from shear stress while providing native ECM cues, successfully extending viable culture to over 7 days. Critically, the model preserves key TME components, including immune cells (CD8+, CD68+) and fibroblasts (ACTA2+), and has been validated for predicting patient-specific drug responses^119^. This work exemplifies how innovative engineering is making high-fidelity slice models a robust platform for clinical applications.

In parallel to preserving existing tissue structures, a powerful “bottom-up” strategy involves the self-assembly of dissociated primary tumor cells into 3D microspheres. These PTCs, typically measuring 40–300 μm in diameter, are formed within a specialized culture medium. Their revolutionary value lies in the creation of a highly biomimetic microenvironment, which preserves the intricate, multi-component interaction network of tumor cells, stromal cells, immune cells, and the native extracellular matrix. Furthermore, they faithfully recapitulate critical aspects of tumor heterogeneity, including driver mutation profiles, histological architectures, such as glandular differentiation patterns, and microenvironmental functions, like tumor–fibroblast signaling crosstalk and immune evasion mechanisms^117^, ^118^, ^119^. In stark contrast to conventional cell lines, which exhibit a clinical concordance rate of merely 40%–60%, and PDX models, which are hampered by protracted generation timelines of 3–6 months and the inherent absence of a human immune component, PTCs demonstrate pronounced superiority in terms of genetic fidelity, rapid generation efficiency (2–7 days), and enhanced clinical predictive accuracy mechanisms^35^^,^^117^, ^118^, ^119^.

The advancement of microtumor has been propelled by a series of technical breakthroughs, with key clinical translation milestones that include: (1) Foundational advancements: The year 2020 marked a foundational milestone with the inaugural establishment of PTC models for gastric, colorectal, and breast cancers. These models facilitated personalized drug screening within a two-week timeframe, achieving a predictive accuracy of 93%^118^. (2) High-throughput capabilities and immune integration: In 2022, Hans Clevers and his team leveraged microfluidic technology to rapidly generate thousands of micro-tumor spheroids, successfully recapitulating the immune microenvironment. In a cohort of seven patients, this approach demonstrated remarkable concordance between its predictions and actual clinical efficacy^120^. Concurrently, another study integrated acoustic assembly technology to construct models incorporating immunosuppressive cells in under 2 min. This platform validated the synergistic efficacy of a cabozantinib and pembrolizumab combination therapy, thereby presenting a novel strategy for cancer immunotherapy^121^. (3) Informing and guiding clinical decision-making: In 2024, a pivotal study established 283 PTC models for non-small cell lung cancer, completing the screening of a thousand potential drugs within just ten days. By dynamically monitoring cell viability and IFNG secretion, the model's predictions of response to PD1 inhibitors achieved an 89% concordance with clinical outcomes^35^. Building on this success, a 2025 extension of the research developed models for soft tissue sarcoma with a 95% generation success rate. Among 47 patients with recurrent and metastatic disease, the platform achieved 100% accuracy in distinguishing chemotherapy responses, offering precise guidance for treatment selection in this challenging patient population^122^. These technological breakthroughs are unequivocally accelerating the clinical translation of microtumor models, fundamentally reshaping the landscape of precision oncology.

#### 2.3.2 Core advantages and critical considerations of microtumors

**Figure 10:**
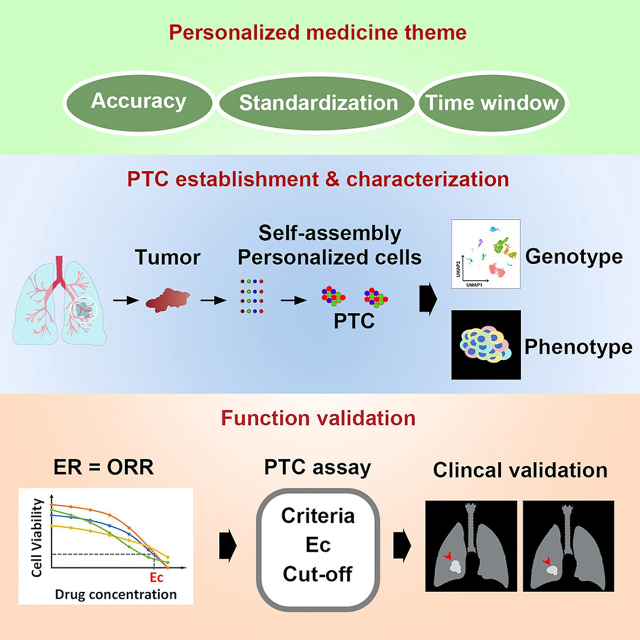
Applications of PTCs in dissecting tumor heterogeneity and predicting therapeutic response^35^. PTCs provide high accuracy, strong standardization, and a short turnaround for personalized medicine (top). Construction and characterization (middle): PTCs are multicellular, self-organized, 3D *in vitro* models derived from autologous patient tumors, preserving the original tumor's phenotypic and genomic features. Functional validation (bottom): Drug screening on PTCs can accurately predict patients' clinical responses to therapy (Reprinted with permission from Ref. 35. Copyright © 2024 Elsevier).

A key advantage of microtumors is their high biological fidelity, which closely recapitulates the essential features of the original patient tumor. This fidelity stems from a rapid self-assembly process that preserves native cellular properties^118^. This high-fidelity biomimicry is critically important and manifests in three distinct areas: (1) Histopathological concordance: The microtumors structurally mirror the patient's own tumor tissue. For instance, a study of 283 non-small cell lung cancer (NSCLC) models established that PTCs accurately reproduced primary tumor histology within days. Models from keratinizing squamous cell carcinomas showed characteristic sharp cellular borders and polygonal morphology, while those from large-cell neuroendocrine carcinomas replicated the native nested architecture^93^. (2) Genotypic and clonal fidelity: These models are a genetic snapshot of the primary tumor, retaining key oncogenic alterations, such as *EGFR* L858R and *ALK* fusions. Single-cell sequencing has confirmed that they also preserve the intratumoral clonal heterogeneity, the complex mixture of different cancer cell subpopulations. This makes them a precise and powerful platform for investigating the emergence of drug resistance^35^. (3) Predictive functional response: By accurately capturing the histological and genetic makeup, PTCs demonstrate clinically relevant drug responses. Their reactions to targeted therapies closely mimic those observed in the patient, establishing them as a pivotal tool for dissecting tumor heterogeneity and guiding individualized treatment decisions (Fig. 10)^35^. In summary, this level of biomimicry excels at capturing the intrinsic heterogeneity of the cancer cell population itself, its architecture, genetics, and resulting drug sensitivity. The next frontier in developing even more sophisticated models lies in incorporating the extrinsic factors, namely the complex interplay with non-cancerous components of the TME.

**Figure 11:**
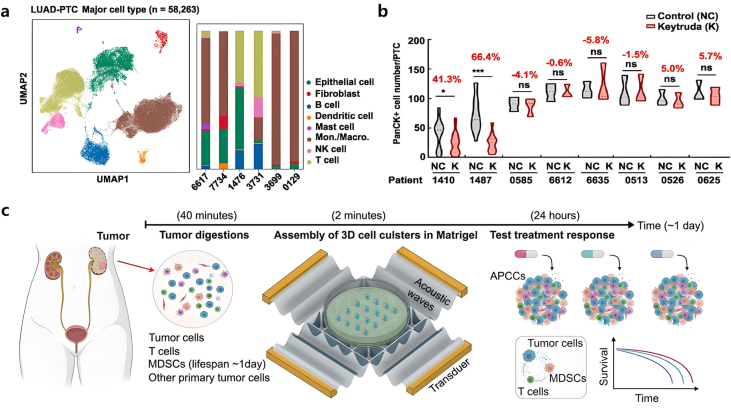
PTCs incorporating an immune microenvironment^121^. (a) Single-cell sequencing identifies tumor endothelial cells, fibroblastic stromal cells, B cells, dendritic cells, monocytes, NK cells, and T cells within NSCLC-PTCs. (b) Effects of the PD1 inhibitor pembrolizumab (Keytruda) after 24 h on PTCs from eight distinct NSCLC patients (Reprinted with permission from Ref. 35. Copyright © 2024 Elsevier). (c) Schematic of rapid PTC construction and drug sensitivity testing *via* acoustic assembly, with retention of tumor-induced immunosuppressive MDSCs (Adapted with permission from Ref. 121. Copyright © 2022 Wiley-VCH).

Building upon their intrinsic cellular fidelity, PTCs offer a more sophisticated advantage by embodying the functional ecosystem of the TME itself. While previous models excel at representing the cancer cell in isolation, these advanced PTCs contain the stromal and immune cells that dictate tumor behavior and, most critically, response to immunotherapy^35^. However, a persistent challenge in reconstructing these models is the differential lifespan of immune cell subsets *ex vivo*. The maintenance of long-term activity varies significantly by cell type. Macrophages and dendritic cells are generally robust, often maintaining viability and phagocytic function within the tumor stroma for 2–3 weeks in microtumor cultures. In contrast, T cells, particularly cytotoxic CD8^+^ T cells, are prone to rapid exhaustion and apoptosis, typically requiring supplementation with exogenous cytokines, such as IL-2 and IL-15, to sustain activity beyond 7–10 days^35^. B cells and NK cells represent the most fragile populations. They are often lost within the first few days of culture unless specific niche factors or stromal support cells are strictly preserved^23^. Microtumors address this by retaining the autologous stromal niche that naturally supports these fragile populations, maintaining their viability and proportions with a high correlation (r > 0.8) to the original tumor's single-cell sequencing profile for the 7–14-day culture period^27^^,^^35^^,^^118^. This is achieved through dissociation methods that preserve a diverse array of non-cancerous cells, which then self-assemble into a miniature tumor–stroma–immune system. This reconstituted TME provides a powerful platform for immuno-oncology research in two key ways: (1) Modeling the immune-active TME for checkpoint inhibition. PTCs can retain essential TME components like tumor-associated fibroblasts (CAFs) and diverse immune populations. For example, NSCLC-derived models containing intratumoral endothelial cells, FAP + fibroblasts, and an immune-cell composition mirroring the primary tumor were used to test immunotherapy. When exposed to the PD1 inhibitor pembrolizumab, 25% of the PTCs exhibited a marked immune response. This demonstrates the model's capacity to functionally validate key immune checkpoint signaling axes, including PD1/PDL1 and CTLA4^35^. While PD1 blockade targets T-cell exhaustion at the effector stage, these models can also evaluate CTLA4 inhibitors, which regulate the priming phase of immune responses. By reproducing the functionality of both checkpoints *in vitro*, microtumors allow for the comprehensive assessment of monotherapies and dual-checkpoint blockade strategies, as evidenced by the secretion of IFNG from preserved CD8^+^ T cells^35^^,^^120^. (2) Modeling the immune-suppressive TME for combination therapies. These models can also replicate the immunosuppressive networks that cause immunotherapy resistance. Using acoustic assembly, researchers rapidly created PTCs that selectively retained myeloid-derived suppressor cells (MDSCs) and reproduced their inhibitory effect on CD8^+^ T cells^99^. This high-fidelity model correctly predicted that a combination of cabozantinib (targeting MDSCs) and pembrolizumab (a checkpoint inhibitor) would yield a superior therapeutic effect, an insight impossible to gain from a model lacking this specific TME component (Fig. 11)^121^. In essence, by capturing the dynamic crosstalk between cancer cells and their surrounding immune and stromal partners, PTCs transcend their role as simple tumor replicas. They become functional preclinical tools that are indispensable for developing and personalizing immunotherapies, a distinct and critical application that sets them apart from simpler 3D models.

Although the high biological fidelity of microtumors is a primary advantage, a key consideration is that cell growth dynamics, spatial organization, and tissue architecture vary significantly depending on the tumor type being modeled. For instance, microtumors derived from CRC often self-organize into glandular or cystic structures that mirror the architecture of the parent tumor, with polarized epithelial layers exhibiting apical-basal polarity and a central lumen, a morphology observed in over 80% of patient-derived CRC organoid lines^61^^,^^120^. In contrast, models from PDAC typically form dense, irregular, and poorly organized cell clusters embedded within a prominent desmoplastic stroma. These models can retain cancer-associated fibroblasts that deposit a dense, collagen I-rich matrix, reflecting the fibrotic and hypovascular nature of PDAC *in vivo*^62^^,^^67^. Glioblastoma microtumors display characteristic cellular dispersion and invasion into the surrounding matrix, with individual cells migrating distances exceeding 500 μm from the tumor core in brain-specific matrix assays, a feature not typically observed in epithelial-derived models^121^.

This inherent variability substantially limits the generalizability of certain conclusions. For example, a conclusion about drug efficacy derived from one model type cannot be directly extrapolated to another. A small-molecule inhibitor might show potent efficacy in a loosely packed CRC microtumor, achieving near-complete target inhibition. However, generalizing this finding to PDAC can lead to inaccurate conclusions. The dense desmoplastic stroma in PDAC microtumors, rich in collagen and CAFs, can create a significant physical barrier. This barrier can reduce the effective concentration of small-molecule drugs like gemcitabine at the tumor core by up to 60%–80% and virtually excludes larger therapeutic antibodies^123^. The same drug could appear ineffective in a PDAC model, not due to a lack of on-target activity, but due to this stromal barrier, a factor that is not present in non-desmoplastic models. Therefore, the interpretation of drug screening results should be contextualized within the specific tumor type and its corresponding microtumor architecture.

This understanding of tumor-specific heterogeneity should directly inform both model design and data interpretation. To model the invasive hallmark of glioblastoma, microtumors should be embedded within a brain-specific ECM hydrogel, such as one composed of hyaluronic acid and laminin, which permits quantitative tracking of cell migration^124^. For PDAC models, dissociation and culture protocols should be optimized to co-preserve not only cancer cells but also pancreatic stellate cells, the precursors to CAFs. The presence of these stromal cells has been shown to confer resistance to standard-of-care chemotherapies like gemcitabine in co-culture models^67^. Regarding data interpretation, microenvironmental cues within the model, such as oxygen gradients, must be considered. As microtumors grow beyond 400–500 μm in diameter, they can develop a hypoxic core, with oxygen partial pressure dropping from atmospheric levels, of around 20%, at the periphery to below 1% in the center^125^. When interpreting a cell viability assay, for example, using an ATP-based method, this baseline level of hypoxia-induced cell death must be distinguished from drug-induced cytotoxicity. An interpretation that does not account for this baseline may lead to an overestimation of drug efficacy, a phenomenon empirically demonstrated where bulk ATP measurements failed to capture the spatial heterogeneity of cell death observed *via* high-content imaging^126^^,^^127^. Therefore, an analysis requires correlating endpoint viability data with spatial information from imaging or specific hypoxic markers such as HIF1A staining. This tailored approach of designing and interpreting models based on the known biology of the tumor of origin is essential to fully utilize the predictive power of microtumors.

#### 2.3.3 Personalized clinical therapy guided by PTCs

**Figure 12:**
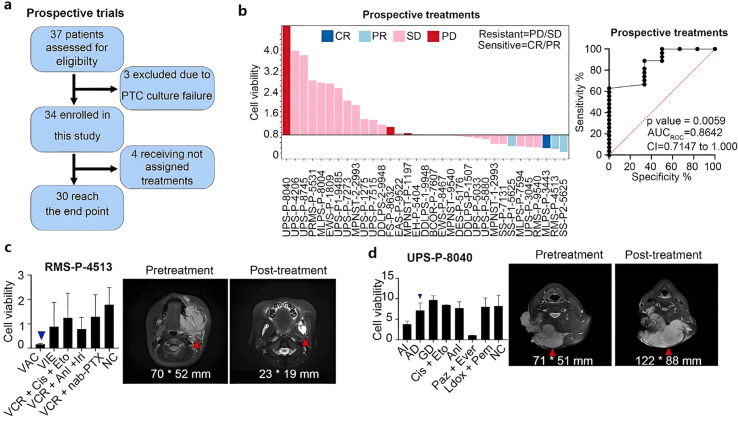
Clinical validation of the predictive performance of PTC models^122^. (a) Prospective clinical trial workflow, among 37 patients, PTC models were successfully established for 34, and 30 received screening-guided therapy. (b) Cell viability readouts from PTC-based drug assays and corresponding prospective clinical outcomes, with analysis of complete response (CR)/partial response (PR) *versus* partial response (PD)/stable disease (SD). (c) The Vincristine + Actinomycin D + Cyclophosphamide (VAC) regimen exhibited marked cytotoxicity against PTCs from patient RMS-P-4513; imaging revealed substantial tumor regression following VAC treatment. (d) The Adriamycin + Dacarbazine (AD) regimen showed no inhibitory effect on PTCs from patient UPS-P-8040. The tumor continued to enlarge under AD therapy (Reprinted with permission from Ref. 122. Copyright © 2025 Cell Press (Elsevier).

The primary clinical utility of PTCs lies in their function as a rapid and predictive platform for personalized medicine. By faithfully recapitulating patient-specific tumor characteristics within a high-throughput format, they can forecast clinical responses to various treatments before they are administered to the patient, thereby minimizing exposure to ineffective therapies. The power of this approach comes from a confluence of factors: a single patient biopsy can generate thousands of individual PTCs, each preserving the original tumor's heterogeneity and microenvironment. This enables the parallel testing of hundreds of drug combinations within a clinically relevant timeframe of 7–14 days^35^^,^^120^. The clinical validity of this model has been demonstrated across multiple cancer types. For example, in NSCLC, a large-scale study using 283 patient-derived models achieved 89% overall concordance in predicting clinical responses for 26 patients. The platform was 98.1% accurate in distinguishing patients who would respond (complete/partial response) from those whose disease would progress, showcasing its direct utility in guiding treatment choices^35^. In CRC, a microfluidics-based system generated thousands of microtumors and completed drug assessments within 13 days, with the model's predictions matching the clinical outcomes in all 7 validated patient cases^120^. In soft-tissue sarcoma (STS), from 254 patient samples, researchers screened hundreds of treatment regimens. In 37 prospectively validated cases, the screening results showed a 78.3% concordance with patient outcomes and were 100% accurate in differentiating responders from non-responders (Fig. 12)^122^. Collectively, these studies establish PTCs as a robust functional tool for precision oncology, enabling clinicians to make evidence-based, individualized treatment decisions by pre-testing therapeutic efficacy *ex vivo*.

#### 2.3.4 Elucidating resistance mechanisms and intervention strategies with PTCs

Beyond immediate treatment guidance, the dynamic and biologically faithful nature of PTCs makes them an invaluable research tool for elucidating the mechanisms of therapeutic resistance and designing novel counterstrategies. Unlike simpler models, PTCs provide a functional 3D system where the complex processes of treatment failure can be observed and manipulated longitudinally^35^. The model's ability to sustain long-term cultures while maintaining clonal heterogeneity is critical. For example, by exposing NSCLC-derived PTCs to the *EGFR* inhibitor gefitinib, researchers could directly observe the emergence of distinct resistant clones. The PTC platform's strength was in enabling the identification of multiple co-existing resistance mechanisms, like *MET* and *HER2* mutations, within a single patient-derived system. This capacity to model the emergence of resistant subclones under therapeutic pressure serves as the critical *in vitro* functional indicator for predicting clinical recurrence. The measurable indicators for this process include the outgrowth kinetics of resistant clones and the loss of initial drug sensitivity over time. Crucially, the platform then served as an immediate testbed to validate a solution: demonstrating that a combination of *EGFR* and *PI3K* inhibitors effectively eradicated these specific resistant populations, thus showcasing the model's end-to-end utility from mechanism discovery to strategy validation^35^. Also, because PTCs retain a functional TME, they serve as a powerful platform for preclinical validation of drugs that can overcome immune-based resistance. Addressing the scarcity of clinical STING pathway agonists, researchers used lung cancer PTCs not just as a biological model, but as a functional screening tool. This platform's key advantage was its physiological relevance, allowing for the successful identification of the existing drug carvedilol as a novel STING activator through a drug repurposing screen. The model then enabled the immediate functional validation of this finding, demonstrating that carvedilol-induced STING activation potentiated the cytotoxicity of chemotherapy within this complex tumor–immune ecosystem^128^. This illustrates the PTC's power to serve as a discovery engine for novel immunomodulatory agents.

### 2.4 A systematic comparison of organoids, organ-on-a-chip, and microtumors

Organoids, organ-on-a-chip, and microtumors are 3D models used for preclinical validation, each offering advantages in biological fidelity, engineering control, and preservation of native microenvironments^21^^,^^22^^,^^64^. However, each platform also faces technical bottlenecks (Table 5)^23^, ^24^, ^25^^,^^34^^,^^35^^,^^37^^,^^46^^,^^58^, ^59^, ^60^, ^61^^,^^66^^,^^67^^,^^78^^,^^79^^,^^87^^,^^88^^,^^90^^,^^99^^,^^113^^,^^118^, ^119^, ^120^, ^121^, ^122^^,^^129^^,^^130^. To address these limitations, organoids and organs-on-chips are being combined into organoid-on-a-chip platforms that recapitulate complex tissue architecture alongside dynamic microenvironments^27^. The capacity of these models for clinical prediction is demonstrated by 75%–89% concordance rates in forecasting clinical outcomes. This performance, alongside growing regulatory acceptance, is accelerating the clinical translation of therapeutic candidates^102^. Nonetheless, the lack of standardization and reliance on sample quality remain barriers to broader adoption. Looking ahead, enhancing standardization and undertaking large-scale prospective studies will be pivotal to clinical translation.

**Table 5:** Head-to-head quantitative and qualitative comparison of advanced 3D tumor models.

Feature	Organoids	Organ-on-a-chip	Microtumors
Generation time	2–4 weeks^25^^,^^58^	Days to weeks (model-dependent)^79^^,^^90^	2–14 days (fastest for clinical use)^35^^,^^120^
Success rate (%)	Around 40%–85% (cancer type-dependent)^37^^,^^46^	High (engineering-driven), but less relevant for patient-specific models unless using primary cells.	Over 90% (highest)^35^^,^^122^
Throughput and cost	High throughput (amenable to high-throughput screening formats). Estimated cost: $500–2000 per patient sample^24^^,^^129^	Low-medium throughput (complex setup). Estimated cost: $100–500 per chip (excluding capital equipment)^113^^,^^130^	High throughput (thousands from one biopsy). Estimated cost: $1000–3000 per patient sample^35^^,^^120^
Morphological/histological	High. Recapitulates glandular polarity, with histology scores showing >90% structural similarity to the primary tumor^59^^,^^60^.	Moderate to high (engineered). Can reconstruct functional tissue barriers, *e*.*g*., blood–brain barrier models achieving transepithelial electrical resistance (TEER) values of >1000 Ω cm^2^, approaching *in vivo* levels^78^^,^^79^.	Highest. Directly preserves native histological subtypes, with >95% histological concordance with the original tumor as assessed by pathologists^35^^,^^119^.
Genomic/epigenetic	High. Retains 80%–90% of somatic mutations and copy number variations for >20 passages^61^.	Variable. Fidelity can be >90% when using patient-derived primary cells but is significantly lower with established cell lines, such as HeLa^34^.	Very high. Concordance rate of >98% for key driver mutations, preserving a clonal architecture highly correlated with the original tumor (correlation coefficient *r* > 0.9).^35^^,^^122^
Cancer hallmarks	Sustains proliferation and evades growth suppressors: Validated by conservation of oncogenes and tumor suppressor mutations^60^^,^^61^. Resisting cell death: Predicts clinical outcomes with 100% sensitivity, 93% specificity, and 88% positive predictive value^66^^,^^67^.	Models invasion and metastasis: Simulates cell migration, intravasation, and extravasation under controlled flow and mechanical stimuli^34^^,^^99^. Activating invasion and metastasis: induces a >3-fold increase in cancer cell invasion under fluidic shear stress. Can form perfusable microvascular networks with >85% vascularization efficiency^88^.	Avoiding immune destruction: Preserved functional CD8^+^ T cells secrete IFNG upon PD1 blockade, with responses observed in 25% of patient models^35^^,^^121^. Retains FAP ^+^ cancer-associated fibroblasts at levels comparable to the original tumor^118^.
TME	Limited. Typically contains <5% CD45^+^ immune cell infiltration, whereas this proportion is often 15%–40% *in vivo*^23^^,^^37^.	Engineered/reconstituted. Can maintain engineered multicellular co-culture systems for >28 days under dynamic perfusion^87^^,^^90^.	Very high. Preserves diverse immune cell infiltrates, with population distributions highly correlated with single-cell sequencing data from the original tumor (*r* > 0.8).^35^^,^^118^^,^^121^

**Figure 13:**
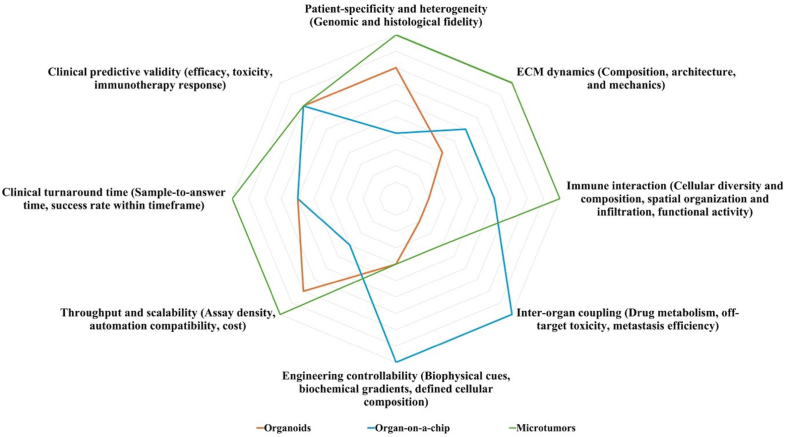
Comparative benchmarking of advanced 3D tumor models. The radar chart provides a semi-quantitative assessment of organoids (orange), organ-on-a-chip (blue), and microtumors (green) across eight key dimensions relevant to preclinical and clinical applications. The radial distance from the center indicates the relative strength of each model in a given dimension. Microtumors demonstrate a dominant profile in categories related to biological fidelity, including patient-specificity, ECM dynamics, and immune interaction, as well as in clinical utility metrics like turnaround time and predictive validity. Organ-on-a-chip exhibits unparalleled performance in engineering controllability and inter-organ coupling, making it distinctly suited for systemic studies. Organoids present a balanced profile, with notable strengths in throughput, scalability, and patient-specificity, positioning them as a core tool for large-scale screening and basic research. The visual comparison underscores that the models are complementary, and selection must be guided by the specific fitness-for-purpose of the intended application.

To systematically benchmark the fitness-for-purpose of these models, a comparative analysis was conducted across eight key performance dimensions (Fig. 13). This analysis reveals distinct and complementary capabilities for each platform. Microtumors demonstrate the highest degree of biological fidelity by preserving the native tumor ecosystem. This model retains not only the original tumor's architecture and clonal heterogeneity but also its intact immune microenvironment, including functional populations of tumor-infiltrating immune cells and stromal components^35^^,^^118^. It is the suitable platform for assessing immunotherapies and TME-mediated drug resistance. In terms of clinical utility, microtumors offer the most rapid generation time of 2–14 days and the highest success rate of over 90%, enabling high-throughput screening of drug combinations within a clinically actionable timeframe^35^^,^^120^^,^^122^. Key technical limitations include a finite *ex vivo* maintenance window and incompatibility with genetic modification or long-term expansion.

In contrast, organ-on-a-chip systems excel in engineering control and the simulation of systemic functions. This platform offers high functional fidelity by precisely recreating dynamic biophysical forces, such as fluid shear stress and mechanical strain, and modeling physiological barriers. Its unique capability for inter-organ coupling allows for the simulation of ADME processes and the assessment of cross-organ toxicity, functions that other models cannot perform^87^^,^^89^^,^^112^. However, the tumor microenvironment in these systems is artificially reconstituted rather than natively preserved. The model is also characterized by low to medium throughput due to its operational complexity and is less suited for generating patient-specific models unless primary cells are used.

Organoids provide a balanced profile, bridging high-throughput screening with high genetic and histological fidelity. They effectively recapitulate the parental tumor's genomic landscape, with studies reporting 80%–90% mutational concordance, making them a robust tool for basic cancer biology and genetic studies^62^. With a generation time of 2–4 weeks and a cancer type-dependent success rate of 40%–85%, organoids are highly scalable for large-scale drug screening^25^^,^^37^^,^^46^^,^^58^. Their primary limitations are an incomplete representation of the TME, as they typically lack a functional immune component and rely on non-native matrices like Matrigel, and an inability to model inter-organ interactions^23^^,^^37^. These models are not substitutive but complementary. The optimal choice is determined by the specific research question or clinical application. Microtumors are best suited for rapid, high-fidelity personalized therapy guidance, particularly for immunotherapies. Organ-on-a-chip is the premier platform for studying systemic pharmacokinetics and toxicity. Organoids remain the model of choice for scalable drug discovery and fundamental research into tumor genetics.

The comparative analysis demonstrates significant complementarity among the three models. No single model is universally optimal; selection depends on its fit-for-purpose application. The utility of each platform is constrained by inherent technical bottlenecks, including the batch-to-batch variability of organoids, the engineered, non-physiological microenvironment in organ-on-a-chip, and the limited *ex vivo* lifespan of microtumors. Addressing these limitations is a prerequisite for their clinical translation. The pathway to clinical adoption relies on standardization, QC, and regulatory qualification. Driven by mandates such as the FDA Modernization Act 2.0, establishing quantifiable acceptance criteria for these models has become a critical challenge in the field. The intrinsic complexity of model fabrication, functional validation, and data interpretation, compounded by the data-intensive nature of high-throughput screening, creates a clear technical demand for AI to enhance analytical efficiency and predictive accuracy.

## 3 Clinical translation of biomimetic 3D tumor models: challenges and strategic solutions

### 3.1 Challenges and technical bottlenecks in clinical translation

The clinical and industrial translation of biomimetic 3D tumor models is contingent on addressing inherent limitations across biology, engineering, and regulation. A primary limitation is the difficulty in preserving native tumor heterogeneity over time. PDOs, while possessing high genetic fidelity, are subject to clonal selection pressures during long-term culture. After extensive passaging, such as >20 passages, clonal architecture can diverge from the primary tumor, resulting in the loss of specific subclones and a decreased representation of the original heterogeneity^93^. This reconstruction bias compromises their predictive accuracy. Conversely, microtumors preserve the native tumor ecosystem but are constrained by a 7–14-day *ex vivo* maintenance window and an inability to be expanded, limiting their use to short-term functional assays^14^^,^^15^^,^^24^. The reliability of immune component maintenance is another critical issue. In reconstituted organoid co-cultures, the functional lifespan of exogenously added T cells is often limited to 48–72 h^29,44^. In contrast, microtumor models retain native, functional immune populations for up to 14 days, as demonstrated by sustained IFNG secretion from CD8^+^ T cells upon checkpoint inhibitor treatment^14^^,^^15^^,^^43^.

From an engineering and manufacturing standpoint, scalability and standardization are major bottlenecks. Current fabrication protocols are low-throughput, with costs estimated at $500–$2000 per patient sample for organoid-based screening, impeding industrial-scale deployment^31^. Reliance on undefined biological matrices like Matrigel introduces significant batch-to-batch variability, stemming from inconsistent concentrations of growth factors such as *EGF* and *TGFB*, which affects experimental reproducibility^3^^,^^53^. Strategic solutions are focused on automation and material science. Microfluidics-based platforms integrated with robotics are being developed to increase throughput, while AI-driven high-content imaging enables automated, quantitative quality control^86^. The development of chemically defined, synthetic hydrogels is critical for eliminating matrix-associated variability^131^.

Logistics and regulations present additional hurdles. Acquisition of fresh, high-quality patient tissue is a primary bottleneck, reflected in the variable establishment success rates of PDOs: >85% for CRC *versus* 42% for pancreatic cancer^28^, ^29^, ^30^^,^^44^. Ethical protocols, including informed consent and institutional review board (IRB) approval, are prerequisites. Although the regulatory landscape is evolving with policies like the FDA Modernization Act 2.0^68^, a key barrier remains: the lack of globally harmonized, specific guidance for qualifying these models for a defined context of use (COU). This ambiguity complicates their integration into IND submissions.

### 3.2 Strategic solutions for addressing the challenges: standardization, quality control, and regulatory qualification

To address the challenges of biological fidelity, engineering reproducibility, and regulatory uncertainty, establishing a framework for standardization, quality control (QC), and qualification is a critical imperative. These challenges define the translational gap between laboratory research and clinical utility. To bridge this gap, a formal validation framework is emerging, driven by legislative mandates, such as the FDA Modernization Act 2.0^118^, dedicated regulatory qualification programs, and industry-led standardization initiatives.

Standardization is a prerequisite for ensuring the comparability and reproducibility of *in vitro* 3D models, which is critical for minimizing experimental variability. This process encompasses two primary domains: biological components and engineering/assay methodologies. For biological components, standardization involves establishing standard operating procedures (SOPs) for cell sourcing and authentication, including protocols for tissue acquisition, transport, digestion, and genetic verification. To address the historical 15%–36% rate of cell line cross-contamination, the International Cell Line Authentication Committee (ICLAC) mandates genetic verification *via* short tandem repeat (STR) profiling^42^. It also necessitates a transition from variable biological matrices. For example, to eliminate batch-to-batch variability from biological matrices like Matrigel, which has inconsistent levels of *EGF* and *TGF-β*, the field is shifting to chemically defined synthetic hydrogels, such as RGD-functionalized PEG gels. Similarly, chemically defined, serum-free media, such as mTeSR™, are replacing variable fetal bovine serum (FBS)^22^^,^^31^^,^^106^. In the engineering and assay domain, standardization requires precise documentation of device parameters, such as chip geometry, materials (like PDMS), and flow rates. Critical parameters are established through inter-laboratory round-robin studies. For example, lung-on-a-chip models require 10%–15% cyclic strain, while liver-on-a-chip models require 0.1–1 dyne/cm^2^ shear stress to maintain cell function^68^. Therefore, it mandates uniform protocols for endpoint measurements, including unified drug concentration gradients, incubation times, viability assays, such as CellTiter-Glo, and data analysis pipelines for metrics, like half maximal inhibitory concentration (IC50) values. The FDA's Comprehensive *in vitro* Proarrhythmia Assay (CiPA) initiative, for instance, standardizes drug cardiotoxicity testing on iPSC-cardiomyocytes across multiple microphysiological systems (MPS) platforms, including unified drug concentrations and incubation times. This provides a clear pathway for the regulatory acceptance of MPS. For high-content imaging, standardized acquisition parameters and image analysis algorithms are essential for consistent data interpretation^42^. For instance, without flat-field correction, fluorescence intensity measurements of identical cells can vary by up to 50% based on their position in the field of view^132^. To address this, the field widely adopts version-controlled analysis pipelines like CellProfiler and validates algorithms against public benchmarks such as the Broad Bioimage Benchmark Collection (BBBC) to ensure robust and comparable data processing^133^.

QC aims to establish a set of acceptance criteria for each model to verify that it meets predefined biological and structural standards before use, thereby demonstrating its fitness-for-purpose. Structural and morphological QC utilizes histology, such as H&E staining, and immunohistochemistry for key biomarkers to confirm the model recapitulates the architectural features of the source tissue. For instance, head and neck squamous cell carcinoma organoids have been shown to express markers such as MKI67^+^/TP63^+^/KRT13^+^, confirming a high degree of molecular concordance with the primary tumor^43^, while microtumor models from NSCLC accurately reproduce the histology of keratinizing squamous cell carcinomas and nested architectures of large-cell neuroendocrine carcinomas^108^. Genomic and transcriptomic QC employs sequencing to compare the model's genetic mutation profile and expression landscape with the original tumor tissue. This high fidelity is a critical QC metric. For instance, studies report that 90% of somatic mutations are conserved in CRC organoids^44^, and an overall mutational profile concordance of 80%–90% is achieved between PDOs and primary tumors^46^. Microtumor models effectively preserve key driver mutations, such as *EGFR* L858R, *ALK* fusions, and clonal heterogeneity^107^. Functional QC ensures the model exhibits core functions consistent with its organ of origin. A key QC standard for a qualified liver organoid or liver-on-a-chip is the stable secretion of albumin and urea^35^, whereas a QC check for an immuno-oncology model involves demonstrating the ability of its immune cells, such as CD8^+^ T cells, to secrete IFNG in response to a PD1 inhibitor, as validated in microtumor models^107^.

The integration of these models into drug development pipelines requires adherence to specific regulatory frameworks. Unlike general research utility, regulatory acceptance demands a formal validation process based on the COU principle. This framework, endorsed by agencies such as the FDA and the European Medicines Agency (EMA), stipulates that a model is qualified not for general replacement of animal testing, but for a specific, well-defined purpose, such as screening for drug-induced liver injury, within a specified range of applicability^15^. To harmonize these standards globally and reduce inter-jurisdictional regulatory friction, the Organization for Economic Co-operation and Development (OECD) issued the Guidance on Good *in vitro* Method Practices (GIVIMP)^134^. This guidance provides the standard for method development, validation, and routine implementation, ensuring data integrity and transferability.

Regulatory bodies have established dedicated programs and policies to operationalize these standards. In the U.S., the FDA's Alternative Methods Working Group was formed to implement the directives of the FDA Modernization Act 2.0^118^, actively developing performance criteria for New Approach Methodologies (NAMs). In Europe, the EMA's 3Rs Working Party, in collaboration with the European Union Reference Laboratory for alternatives to animal testing (EURL ECVAM), oversees the qualification of non-animal testing methods to operationalize the 3Rs principle (Replacement, Reduction, and Refinement) and has formally accepted 3D cell systems^121^. These initiatives, alongside guidelines from China's NMPA^15^, are collectively fostering a favorable global environment for the adoption of NAMs over animal testing (Table 6)^100^^,^^134^, ^135^, ^136^, ^137^, ^138^, ^139^, ^140^, ^141^. Industry consortia and specific case studies provide the evidentiary basis for this transition. The International Consortium for Innovation and Quality in Pharmaceutical Development (IQ), for instance, conducts multi-center ring trials to systematically evaluate the performance, reproducibility, and predictive power of platforms such as kidney and gut chips. A large-scale evaluation of the Emulate, Inc. liver-chip platform, involving 870 chips tested with 27 drugs, demonstrated a sensitivity of 87% and specificity of 100% in detecting drug-induced liver injury for compounds that had previously passed animal testing, providing core evidence for COU qualification^99^. A landmark milestone was the FDA's validation of an IND application for Sanofi's antibody therapeutic TNT005 using data derived solely from an organ-on-a-chip model, marking a significant precedent for regulatory adoption^15^. This decision has encouraged companies in China, such as Qilu Pharmaceutical and Yimiao Shenzhou, to follow a similar model for their IND filings^99^^,^^100^. Large-scale, multi-center prospective clinical trials are providing critical validation by comparing model predictions with actual patient outcomes. Published data have demonstrated high predictive performance, including a 100% NPV and 100% sensitivity with 93% specificity for PDOs from gastrointestinal cancers in predicting clinical outcomes^50^^,^^59^, 89% concordance for NSCLC microtumor models^107^, and 100% accuracy in distinguishing responders from non-responders in soft-tissue sarcoma models^122^. These findings offer quantitative evidence to support the clinical adoption of such platforms.

**Table 6:** Evolution of key policies and guidelines for advanced *in vitro* 3D tumor models in key regions.

Region/organization	Timeline	Policy/initiative	Regulatory impact
Global/OECD	2018	Guidance on GIVIMP	Established international technical standards for method development and validation to ensure laboratory reproducibility and regulatory acceptance^134^.
United States	2012–Present	Tissue chip program and alternative methods Working group.	Laid the groundwork through the tissue chip initiatives. The Working group advances the COU qualification pathway and defines performance criteria for NAMs^100^^,^^135^, ^136^, ^137^.
	2022	FDA modernization Act 2.0	A legislative landmark authorizing the use of data from non-animal methods to support IND applications, officially removing the mandatory requirement for animal testing^138^.
	2025	Centers for Disease control and Prevention (CDC) internal reform on non-human primate (NHP) research	Ends NHP experimentation by year-end, accelerating the urgent transition to high-fidelity human-specific models.
European Union	2010s–Present	3Rs Working Party and EURL ECVAM	Defines the pathway for regulatory qualification of NAMs in accordance with the 3Rs principle^100^^,^^135^^,^^136^.
	2023–Present	EMA's work plan and standardization roadmap	Focuses on developing detailed standardization roadmaps and advancing the implementation of qualified methods^139^.
China	2021–2024	Guidelines on gene therapy and organoid consensuses	Released guidelines for non-clinical research and expert consensuses on tumor organoids, establishing initial standards for model construction and QC^140^^,^^141^.

## 4 Conclusions and perspectives

**Figure 14:**
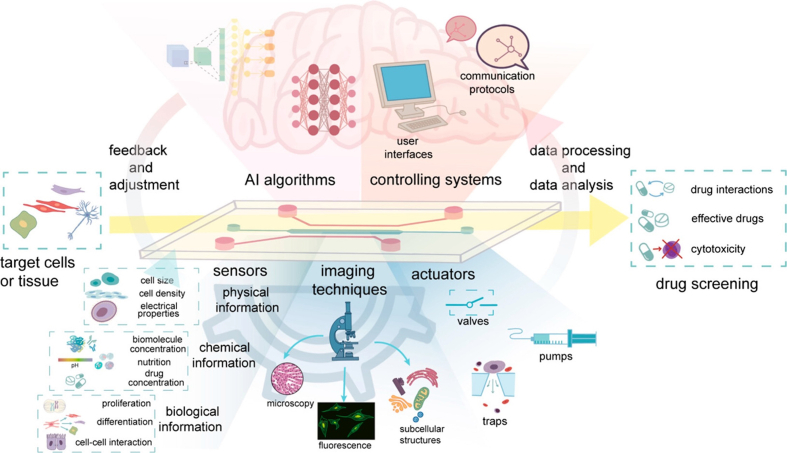
Framework of an intelligent organ-on-a-chip system for automated drug screening. This schematic illustrates an AI-integrated, closed-loop organ-on-a-chip platform^142^. (1) Target cells or tissues are cultured within the organ-on-a-chip. A multi-modal sensing and imaging module acquires real-time data. Sensors monitor physical parameters (cell size and density) and chemical parameters (pH and drug concentration). Imaging technologies, such as microscopy and fluorescence imaging, capture key biological data (*e*.*g*., cell proliferation, differentiation, subcellular structures). Actuators, such as micropumps and valves, precisely regulate the on-chip microenvironment. (2) The acquired multi-dimensional data are transmitted to a central control system. AI algorithms, such as neural networks, process and analyze these data to evaluate drug responses, such as identifying effective compounds, analyzing drug interactions, and detecting cytotoxicity. (3) The AI analysis results generate real-time commands that are relayed back to the system's actuators. These commands dynamically adjust experimental parameters, such as drug dosage and flow rate, enabling autonomous optimization and adjustment of the experimental process (Reprinted with permission from Ref. 142. Copyright © 2024 American Chemical Society).

Advanced 3D biomimetic models, organoids, organs-on-chips, and microtumors serve as alternatives to animal testing and significantly advance new drug development by complying with the 3Rs principle. Organoids provide high genomic fidelity and scalability for high-throughput screening but are limited by the absence of a native immune system and batch-dependent variability. Organ-on-a-chip platforms excel in reconstructing physiological barriers and simulating systemic interactions, yet their application is constrained by engineering complexity and lower throughput. Microtumors uniquely preserve the native immune microenvironment for immunotherapy evaluation, although they face challenges regarding their short *ex vivo* lifespan and inability to undergo expansion. Despite these technical advantages, the widespread industrial adoption of these models is currently impeded by a fundamental lack of standardization in culture protocols and quality control. Addressing these standardization issues requires substantial policy support and the development of harmonized regulatory guidelines. Furthermore, the integration of AI offers a promising direction to overcome data complexity and facilitate the standardization of these advanced systems for routine clinical application (Fig. 14)^142^.

In the imaging pipeline, AI is used to extract quantitative biological information from high-content microscopy images. Imaging workflows based on convolutional neural networks (CNNs) enable automated, multi-level 3D segmentation of nuclei, cells, and whole organoids^143^^,^^144^. For instance, tools such as 3DCellScope utilize pre-trained deep learning models, such as DeepStar3D, for nuclei segmentation, achieving high F1IoU50 scores across diverse datasets and reducing processing time by 20%–70% compared to other networks like Cellos and AnyStar^13^. Other approaches leverage architectures such as YOLOv4 to identify and enumerate rare circulating tumor cells (CTCs) from bright-field image cytometry of peripheral blood with high throughput (50,000 cells per minute) and high accuracy^144^^,^^145^. These pipelines automatically extract hundreds of morphological and topological features, such as volume, shape, and cell-to-neighborhood relationships, from vast image libraries, creating high-dimensional morphological fingerprints for each biological sample and converting subjective visual observation into objective, quantitative data^143^^,^^144^.

In the omics pipeline, AI is employed to process the large-scale, high-dimensional molecular data generated from these models. AI algorithms can identify patterns that traditional analyses cannot detect due to data complexity and heterogeneity. For example, in single-cell transcriptomics analysis, machine learning workflows like devCellPy enable automated annotation of complex cellular hierarchies, accurately identifying cell types across developmental stages and successfully constructing a mouse cardiac developmental atlas^146^. For multi-modal data integration, multi-task deep neural networks such as UnitedNet can fuse data from different omics layers, such as genomics and transcriptomics, to reveal cell-type-specific, cross-modal feature correlations, providing a more comprehensive characterization of a cell's state^124^.

The core function of the integrated decision-support pipeline is to fuse data from the preceding pipelines to support clinical decisions. Instead of analyzing morphology or gene expression in isolation, AI models correlate them. For instance, a model can learn that a specific morphological fingerprint (from imaging) is strongly associated with a particular gene expression profile or drug-resistance mutation (from omics). This morpho-molecular linkage constitutes a multi-modal biomarker. Based on this fused analysis, AI can build high-accuracy predictive models. For instance, one study used drug-screening data from colorectal and bladder cancer organoids, which included both morphological and genomic data, to identify reliable drug biomarkers and accurately predict patient clinical drug responses *via* a network-based machine learning approach^146^. Similarly, by analyzing images from a glioblastoma-on-a-chip, a CNN model could accurately predict key parameters governing cell behavior, such as proliferation and invasion, offering a potential tool for assessing patient-specific tumor evolution^147^. Moreover, by integrating imaging and metabolomics data from organs-on-chips, such as liver and kidney chips, AI can predict drug toxicity in real-time and infer underlying mechanisms, allowing for the optimization of drug candidates at early research and development stages and reducing the risk of clinical trial failure^89^^,^^148^. However, AI implementation remains at a preliminary stage, currently constrained by the absence of standardized evaluation metrics and consensus protocols essential for cross-platform reproducibility.

In conclusion, organoids, organs-on-chips, and microtumors are evolving from experimental models into essential platforms for precision medicine. This transformation is underpinned by the modernization of regulatory science. The systematic integration of high-fidelity biological modeling with AI-driven analytics significantly refines the prediction of patient-specific drug efficacy and systemic safety. By establishing a rigorous evidence-based bridge between preclinical research and clinical outcomes, this synergistic approach supports the design of multi-center trials to validate predictive utility. These advancements will guide the development of future regulatory policies and accelerate the delivery of personalized therapeutic regimens to patients.

## Author contributions

Mengjiao Xia: Formal analysis, Investigation, Resources, Writing - Original Draft, Writing - Review & Editing. Shuqi Wang: Formal analysis, Funding acquisition, Project administration, Supervision, Writing - Review & Editing. Guohua Wu, Di Wu, Wenqi Hu, and Hongbin Deng: Writing - Review & Editing.

## Data availability

No new data were created or analyzed in this study. Data sharing is not applicable to this article.

## Declaration of generative AI and AI-assisted technologies in the writing process

During the preparation of this work, the authors used DeepSeek-R1 (DeepSeek-AI) to improve the readability and language of the manuscript. After using this tool, the authors reviewed and edited the content as needed and take full responsibility for the content of the publication.

## Conflicts of interest

The authors declare no conflicts of interest.
